# Small RNA mediated gradual control of lipopolysaccharide biosynthesis affects antibiotic resistance in *Helicobacter pylori*

**DOI:** 10.1038/s41467-021-24689-2

**Published:** 2021-07-21

**Authors:** Sandy R. Pernitzsch, Mona Alzheimer, Belinda U. Bremer, Marie Robbe-Saule, Hilde De Reuse, Cynthia M. Sharma

**Affiliations:** 1 Department of Molecular Infection Biology II, Institute of Molecular Infection Biology (IMIB), University of Würzburg, Würzburg, Germany; 2 Institut Pasteur, Helicobacter Pathogenesis Unit, Microbiology Department, Paris, France; 3 Present Address: SCIGRAPHIX - Scientific Illustrations, Würzburg, Germany; 4 Present Address: Equipe Atip-Avenir, Centre Régional de Recherche en Cancérologie Nantes/Angers, INSERM U892, CNRS U6299, Angers, France

**Keywords:** Bacterial immune evasion, Bacterial genetics, Pathogens, Small RNAs

## Abstract

The small, regulatory RNA RepG (Regulator of polymeric G-repeats) regulates the expression of the chemotaxis receptor TlpB in *Helicobacter pylori* by targeting a variable G-repeat in the *tlpB* mRNA leader. Here, we show that RepG additionally controls lipopolysaccharide (LPS) phase variation by also modulating the expression of a gene (*hp0102*) that is co-transcribed with *tlpB*. The *hp0102* gene encodes a glycosyltransferase required for LPS O-chain biosynthesis and in vivo colonization of the mouse stomach. The G-repeat length defines a gradual (rather than ON/OFF) control of LPS biosynthesis by RepG, and leads to gradual resistance to a membrane-targeting antibiotic. Thus, RepG-mediated modulation of LPS structure might impact host immune recognition and antibiotic sensitivity, thereby helping *H. pylori* to adapt and persist in the host.

## Abstract (web summary)

The small RNA RepG modulates expression of chemotaxis receptor TlpB in *Helicobacter pylori* by targeting a length-variable G-repeat in the *tlpB* mRNA. Here, Pernitzsch et al. show that RepG also gradually controls lipopolysaccharide biosynthesis, antibiotic susceptibility, and in-vivo colonization of the stomach, by regulating a gene that is co-transcribed with *tlpB*.

## Introduction

Lipopolysaccharide (LPS) is essential for the physiological integrity and functionality of the outer membrane of most Gram-negative bacteria. As a main surface antigen, it also plays an important role in the interaction between bacterial pathogens and their host. LPS is one of the most potent stimulators of the host immune system and its recognition is essential for the host organism to clear bacterial infections^1^. During the course of infection, many pathogens modify their LPS synthesis and structure to adapt to diverse microenvironments and evade recognition by the host’s immune system^2^.

*Helicobacter pylori* is a Gram-negative pathogen that colonizes the stomach of 50% of the world’s population, which can lead to peptic ulcer disease and gastric cancer^3^. *H. pylori* produces a highly modified LPS, unique in both its structure and function, that is essential for establishing colonization and persistence within the human stomach^4–6^. Like in most Gram-negative bacteria, *H. pylori* LPS is composed of lipid A, a core oligosaccharide, and the hypervariable O-specific polysaccharide repeats (O-chain)^7^. *H. pylori* LPS lacks a canonical inner and outer core organization, and was recently reported to have a short core and a longer O-antigen domain, including structures that were previously assigned to the outer core^8^. This long O-antigen comprises a trisaccharide (Trio), a glucan, a DD-heptan, and terminal Lewis antigens. Despite its important roles in immune evasion and persistence, a complete picture of the LPS biosynthesis pathway in *H. pylori* is only being revealed stepwise^4,9,10^. This might be due to the dispersed genomic locations of LPS genes throughout the *H. pylori* genome.

Compared to enterobacterial LPS, *H. pylori* LPS has a reduced immunostimulatory effect (~1,000-fold reduced) due to an under-acetylated and dephosphorylated lipid A^2^. Moreover, the *H. pylori* O-chain contains fucosylated oligosaccharides that mimic the structure of mammalian histo-blood group antigens, including Lewis x/y antigens^2,5^. Both the low endotoxicity of the lipid A and the molecular mimicry of human Lewis antigens contribute to *H. pylori* host immune surveillance and establishment of long-lasting infection^7,11,12^. *H. pylori* LPS is highly variable^13^. Phase-variable ON/OFF expression of LPS biosynthetic genes, such as Lewis antigen-producing fucosyltransferases, has been shown to increase the diversity of LPS phenotypes, thereby enabling *H. pylori* to adapt to its individual host and/or changing environments in the gastric mucosa during infection^14,15^. Different *H. pylori* clinical isolates were found to extensively alter their Lewis antigen expression in vivo, probably reflecting bacterial adaptation to intra-individual host environments^16^.

Hypervariable simple sequence repeats (SSRs) are a major source of so-called phase variation, which facilitates adaptation to changing environments and immune escape of pathogens^17,18^. Length variation of SSRs occurring during replication can either affect translation through the introduction of frameshift mutations (intragenic) or transcription (intergenic) by changing the spacing of promoter elements or transcription factor binding sites^17^. We previously uncovered that SSRs can also serve as targeting sites for small regulatory RNAs (sRNAs), an important class of post-transcriptional regulators controlling gene expression in response to various stress conditions or during infection^19,20^. We previously demonstrated that the conserved and abundant *H. pylori* sRNA RepG (Regulator of polymeric G-repeats) directly base-pairs with its C/U-rich terminator loop to a phase-variable homopolymeric G-repeat in the 5′ untranslated region (UTR) of the *tlpB* mRNA, encoding the chemotaxis receptor TlpB^21^. The length of this SSR determines the outcome of post-transcriptional control (activation/repression) of TlpB by RepG and thereby gradually modulates *tlpB* expression. Although TlpB is assumed to play a role in quorum sensing, biofilm dispersal, acid-/urea-sensing, and pH-taxis^22–25^, its role in host colonization by *H. pylori* is controversial^23,26,27^. Thus, the biological rationale of controlling TlpB via a variable SSR remained unclear.

Using sequence conservation analysis, we noticed that *H. pylori tlpB* is typically encoded upstream of *hp0102*, a gene with previously unknown function. Here, we show that the encoded HP0102 protein is required for LPS O-chain production as well as Lewis x antigen display and is essential for murine stomach colonization. Furthermore, we show that Δ*hp0102* mutants display an increased sensitivity to high-salt stress and antibiotics treatment. Our study demonstrates that RepG co-regulates expression of *tlpB* and *hp0102*, which are transcribed in a bicistronic mRNA, and that the length of the homopolymeric G-repeat in the *tlpB-hp0102* leader determines the outcome of RepG-mediated control of *tlpB-hp0102* mRNA. The post-transcriptional regulation of *hp0102* expression by antisense base-pairing of a sRNA to a variable SSR allows for a gradual modulation of LPS biosynthesis as well as Lewis x antigen display, a more scalable mechanism than the previously reported ON/OFF control. In turn, the fine-tuning of LPS O-chain expression also mediates gradual resistance of *H. pylori* to a membrane-targeting antibiotic.

## Results

### Expression of the *tlpB*-*hp0102* operon is regulated by RepG

**Fig. 1:**
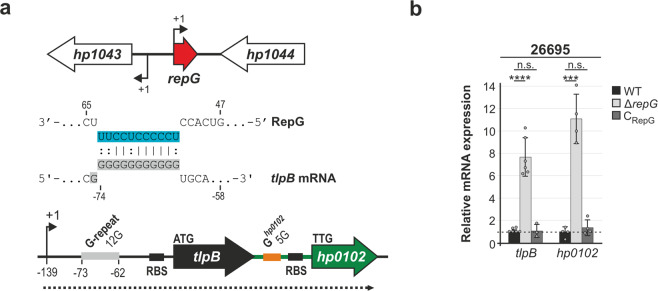
RepG sRNA represses expression of the *tlpB*-*hp0102* mRNA. **a** In *H. pylori* strain 26695, RepG sRNA is transcribed from the intergenic region between *hp1043* and *hp1044*, encoding an orphan response regulator and a protein of unknown function. The C/U-rich terminator loop of RepG (blue) binds to a homopolymeric G-repeat (12G, light gray) in the 5′ UTR of the bicistronic *tlpB*-*hp0102* mRNA (dotted line), encoding a chemotaxis receptor and a protein of unknown function^21^. Transcriptional start sites (TSS, +1; ref. ^28^) and ribosome binding sites (RBS) are indicated by arrows and black bars, respectively. Numbers designate the distance to the *tlpB* start codon. A G-rich sequence (G^*hp0102*^, orange) was identified in the *tlpB*-*hp0102* IGR. **b** RT-qPCR analysis of RepG-dependent regulation of *tlpB* and *hp0102* in *H. pylori* strain 26695 wildtype (WT), *repG* deletion (∆*repG*), and complementation (C_RepG_) mutants. Wild-type mRNA levels of each gene were set to 1, and relative mRNA levels in mutants are shown as bars. Values are shown as mean ± standard deviations (s.d.) for *n* = 6 (*tlpB*; exception C_RepG_ *n* = 3) and *n* = 4 (*hp0102*) biologically independent experiments. ****—highly significant, *p*-value < 0.0001; ***—highly significant, *p*-value < 0.001; n.s.—not significant; Student’s *t*-test, two-tailed. Source data underlying (**b**) is provided as a Source data file.

The *H. pylori* RepG sRNA directly interacts with a 12G-repeat in the 5′ UTR of *tlpB* mRNA and thereby post-transcriptionally represses *tlpB* in strain 26695 (Fig. 1a, ref. ^21^). RepG is highly conserved among different *H. pylori* strains, and homologs are also present in the related gastric *Helicobacter* species, *H. acinonychis*, *H. cetorum*, and *H. mustelae*^21^. Our previous dRNA-seq study^28^ suggested *tlpB* is transcribed as a bicistronic mRNA together with its downstream gene *hp0102*, which we could validate by RT-PCR analysis (Supplementary Fig. 1a, b). We found a highly conserved genetic organization with adjacent *tlpB* and *hp0102* genes in 86 *H. pylori* genomes (Supplementary Fig. 1c). Notably, in the closely related *H. acinonychis* and *H. cetorum* species, the *tlpB* and *hp0102* homologous genes are also adjacent and preceded by a G-repeat sequence. The conserved genetic organization of *tlpB* and *hp0102* was suggestive of their co-expression and co-regulation by RepG.

RT-qPCR analysis showed about eight- and ten-fold increase in mRNA levels of the *tlpB* and *hp0102* genes, respectively, in the Δ*repG* mutant when compared to the wildtype (WT) of *H. pylori* strain 26695 (Fig. 1b). This co-regulation is in line with *tlpB* and *hp0102* being encoded in an operon. Complementation of the Δ*repG* mutant with ectopically expressed RepG under control of its native promoter from the neutral *rdxA* locus (C_RepG_) restored wild-type mRNA levels of both genes. This indicates that not only *tlpB*, but also *hp0102* is repressed by RepG in *H. pylori* strain 26695.

### RepG represses HP0102 by targeting an upstream G-repeat

**Fig. 2:**
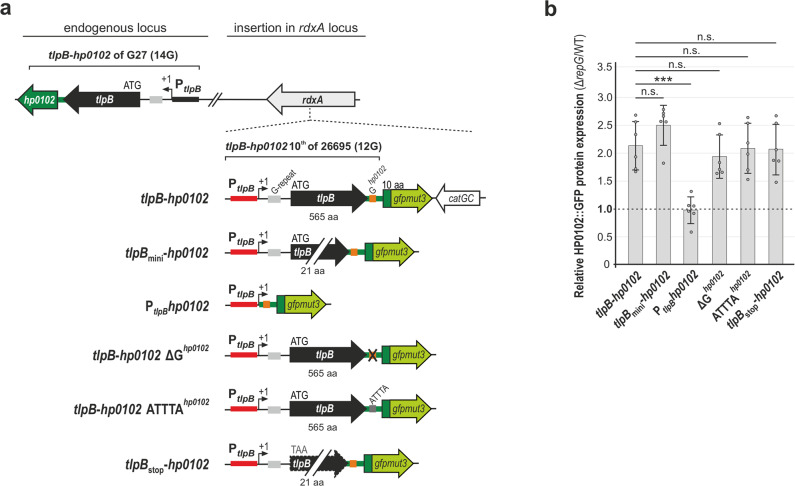
RepG represses HP0102 protein expression through targeting the G-repeat upstream of *tlpB-hp0102*. **a** Schematic representation of translational *hp0102*::*gfpmut3* reporter fusion constructs originating from *H. pylori* strain 26695 (12G) that were integrated into the *rdxA* locus in *H. pylori* G27 wildtype and Δ*repG*. The endogenous *tlpB-hp0102* operon in G27 carries a 14G repeat. Red bar: *tlpB* promoter region; gray: G-repeat upstream of *tlpB*; black: *tlpB* coding region; orange: G-*repeat* upstream of *hp0102*; dark green: HP0102 coding region; light green: *gfpmut3* coding sequence. Arrows indicate the transcriptional start site. **b** *H. pylori* G27 WT and Δ*repG* strains that carry the indicated GFP reporter fusions were grown to exponential phase and HP0102::GFP protein levels were determined by western blot analysis. The relative HP0102::GFP protein levels in the Δ*repG* mutant (vs. the respective wild-type background) are indicated by bars with corresponding s.d. (*n* = 6 biologically independent experiments; corresponding western blot representative is shown in Supplementary Fig. 3). ***—highly significant, *p*-value < 0.001; n.s.—not significant; Student’s *t*-test, two-tailed. Source data underlying (**b**) is provided as a Source data file.

To investigate whether RepG also controls HP0102 protein synthesis, we used a translational *gfpmut3* reporter system established in strain G27^21^. Here, the first ten amino acids of the 26695 *hp0102* coding region were fused to *gfpmut3* and introduced together with *tlpB*, including native promoter (P_*tlpB*_) and 5′ UTR (12G), into the *rdxA* locus of G27 (*tlpB*-*hp0102*, Fig. 2a). HP0102::GFP protein levels were increased about two-fold upon *repG* deletion, demonstrating that RepG represses expression of HP0102 (Fig. 2b and Supplementary Fig. 3). Full-length *tlpB* expression seemed to be dispensable for RepG-mediated HP0102 regulation because HP0102::GFP expression was also increased more than two-fold in *H. pylori* G27 carrying a *tlpB*_mini_-*hp0102* reporter fusion upon *repG* deletion. The latter comprises a non-functional 21 codon-long *tlpB* mini-gene, in which the middle region (544 codons) was deleted (Fig. 2a, b).

The *hp0102* TTG start codon in the 30-nt-long intergenic region (IGR) between *tlpB* and *hp0102* is preceded by a conserved potential AAGGGT ribosome binding site (RBS) (Fig. 1a and Supplementary Fig. 4b), suggesting co-transcription but not necessarily co-translation of both genes. A short, variable G-rich sequence (G^*hp0102*^, 3–6G) is found upstream of the *hp0102* RBS, which might also be targeted by the C/U-rich loop of RepG sRNA (Supplementary Fig. 4c). To investigate whether the G^*hp0102*^-repeat is involved in RepG-mediated HP0102 regulation, the 30-nt-long *tlpB*-*hp0102* IGR followed by the *hp0102* 10^th^::*gfpmut3* coding region were fused to the *tlpB* promoter (P_*tlpB*_*hp0102*, Fig. 2a). Equally expressed HP0102::GFP fusion protein levels in the wildtype and the Δ*repG* mutant carrying the P_*tlpB*_*hp0102* reporter fusion indicated that the G^*hp0102*^-repeat is not sufficient for RepG-mediated HP0102 expression control (Fig. 2b and Supplementary Fig. 3). While slight differences in basal expression levels of HP0102::GFP were observed in the wildtype, neither deletion of the G^*hp0102*^-repeat (ΔG^*hp0102*^) nor its exchange to an ATTTA-stretch (ATTTA^*hp0102*^) did significantly affect RepG-mediated repression of HP0102::GFP (Fig. 2b and Supplementary Fig. 3).

We had previously shown that RepG regulates *tlpB* expression mainly at the level of translation^21^. Therefore, although harboring a separate RBS, HP0102 expression control through RepG might still be coupled to *tlpB* mRNA translation. Replacement of the *tlpB* start codon by a stop codon in the *tlpB*_mini_-*hp0102* reporter fusion (*tlpB*_stop_-*hp0102*, Fig. 2a) neither affected HP0102::GFP protein expression nor RepG-mediated repression thereof (Fig. 2b and Supplementary Fig. 3). This shows that *hp0102* translation and regulation is not directly influenced by *tlpB* translation. Taken together, these data demonstrate that targeting of the homopolymeric G-repeat in the *tlpB* mRNA leader by the RepG sRNA mediates a coordinated regulation of the *tlpB*-*hp0102* operon, at the transcript and protein level.

### HP0102 is required for mouse stomach colonization

Variable SSRs are often associated with genes important for host-pathogen interactions^18,29^. To investigate whether RepG sRNA and/or its targets, *tlpB* and *hp0102*, contribute to virulence and/or survival, we infected mice with either wildtype or non-polar deletion mutant strains of the mouse-adapted strain *H. pylori* X47-2AL (as in ref. ^30^). While the syntenic organization of the *tlpB*-*hp0102* operon in strain X47-2AL is the same as in strain 26695 (Supplementary Fig. 1c), the G-repeat in the *tlpB* mRNA leader is only 7Gs long in X47-2AL compared to 12Gs in 26695 (Supplementary Fig. 4a). An approximately two-fold repression of *tlpB* through RepG was observed in strain X47-2AL compared to an about five-fold repression in strain 26695 (Supplementary Fig. 2). This is in line with the previously described strain-specific, RepG-mediated *tlpB* regulation depending on G-repeat length^21^.

**Fig. 3:**
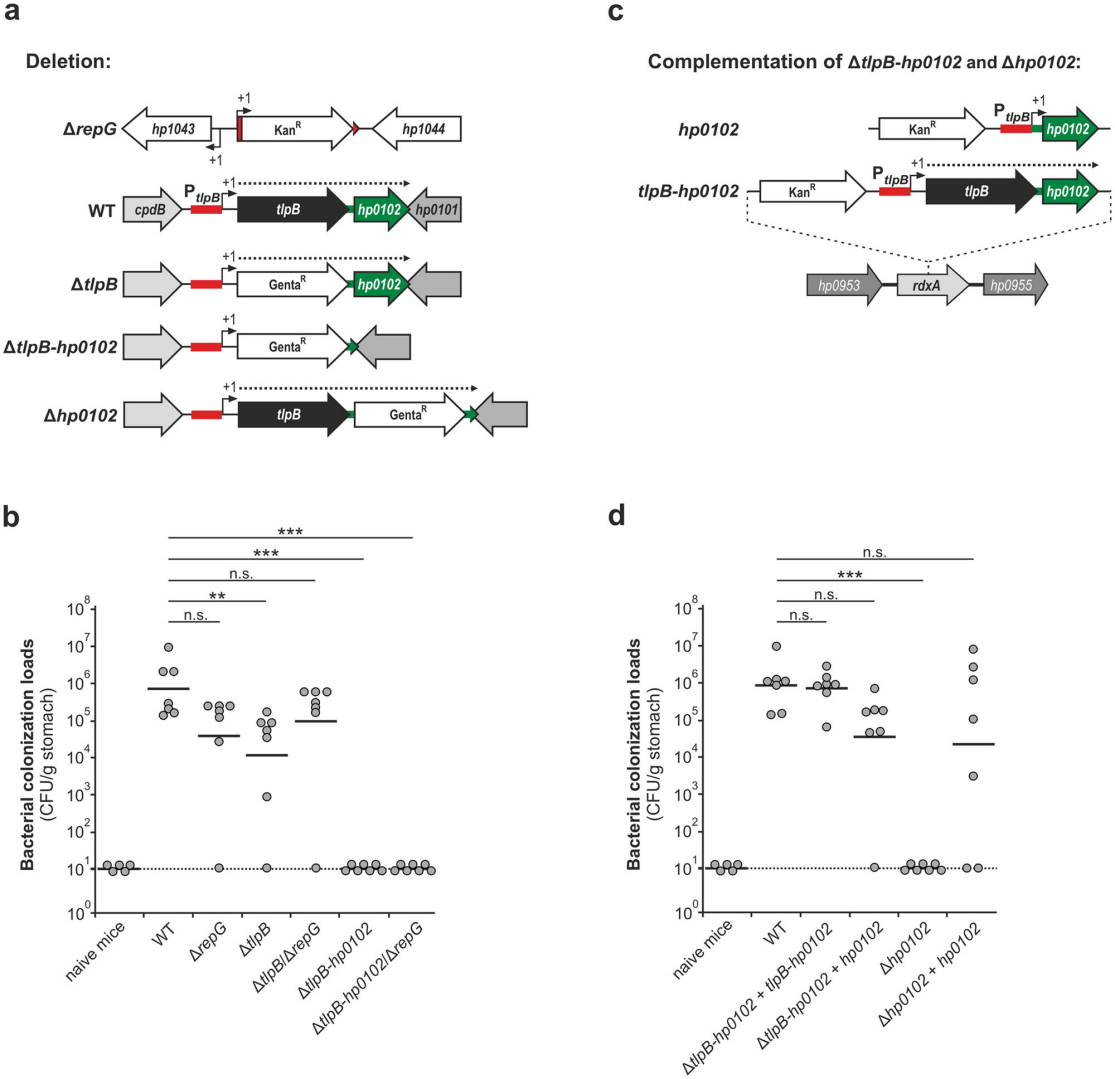
HP0102 is essential for mice stomach colonization with *H. pylori* strain X47-2AL. **a**, **c** Schematic representation of the construction of *H. pylori* X47-2AL ∆*repG* and ∆*tlpB/hp0102* deletion mutant and complementation strains. TSS are denoted as +1 (black arrows). The *repG* gene and the *tlpB* promoter (P_*tlpB*_) are shown in red. The *hp0102* gene is shown in green. **b**, **d** About 10^8^ bacteria of *H. pylori* X47-2AL WT or indicated mutant strains were orogastrically administered to NMRI Swiss mice. As a control, mice were infected with peptone broth only. Four weeks post infection, mice were sacrificed and colony-forming units (CFUs) per gram of stomach weight were calculated by serial dilutions and plating assays. Each circle indicates the colonization titer in the stomach of a single mouse. The horizontal bars represent the geometric mean for each group of data (*n* = 5/4 non-infected control animals for panel b/d, respectively; *n* = 7 for mice infected with indicated *H. pylori* strains). ***—highly significant, *p*-value < 0.001; **—very significant, *p*-value < 0.01; n.s.—not significant; Mann–Whitney test (Prism), two-tailed. Source data underlying (**b**, **d**) are provided as a Source data file.

While no significant change was observed for the Δ*repG* or Δ*tlpB*/Δ*repG* double deletion mutants, in vivo infection studies revealed that the *H. pylori* X47-2AL Δ*tlpB* single mutant is slightly attenuated (~1.5-log lower CFU/g of stomach ) in its ability to colonize the murine stomach compared to the wildtype (Fig. 3b). This minor effect suggests that both RepG and TlpB are largely dispensable for *H. pylori* mouse stomach colonization. In contrast, no bacteria could be recovered from the stomachs of mice infected with the ∆*tlpB-hp0102*, Δ*tlpB*-*hp0102*/∆*repG*, and ∆*hp0102* deletion mutants (Fig. 3b, d), indicating that *hp0102* is essential for *H. pylori* to colonize mice. Accordingly, stomach colonization was restored in independent infection experiments, in which either the single Δ*hp0102* or double Δ*tlpB*-*hp0102* mutants were complemented with either the whole *tlpB-hp0102* operon or only *hp0102* expressed from the *tlpB* promoter (P_*tlpB*_) in the *rdxA* locus (Fig. 3c, d). A slightly reduced colonization level compared to wildtype (WT) was still observed in the Δ*hp0102* + *hp0102* strain, which might be due to altered *hp0102* expression, e.g., caused by the fusion of the *tlpB-hp0102* intergenic region (IGR) to P_*tlpB*_. Overall, our in vivo infection studies identified *hp0102* as an essential factor for colonization of the murine stomach by *H. pylori*. Therefore, we aimed to explore a potential link between the variable G-repeat and host colonization that would rely on the function of HP0102.

### HP0102 is involved in O-chain biosynthesis

**Fig. 4:**
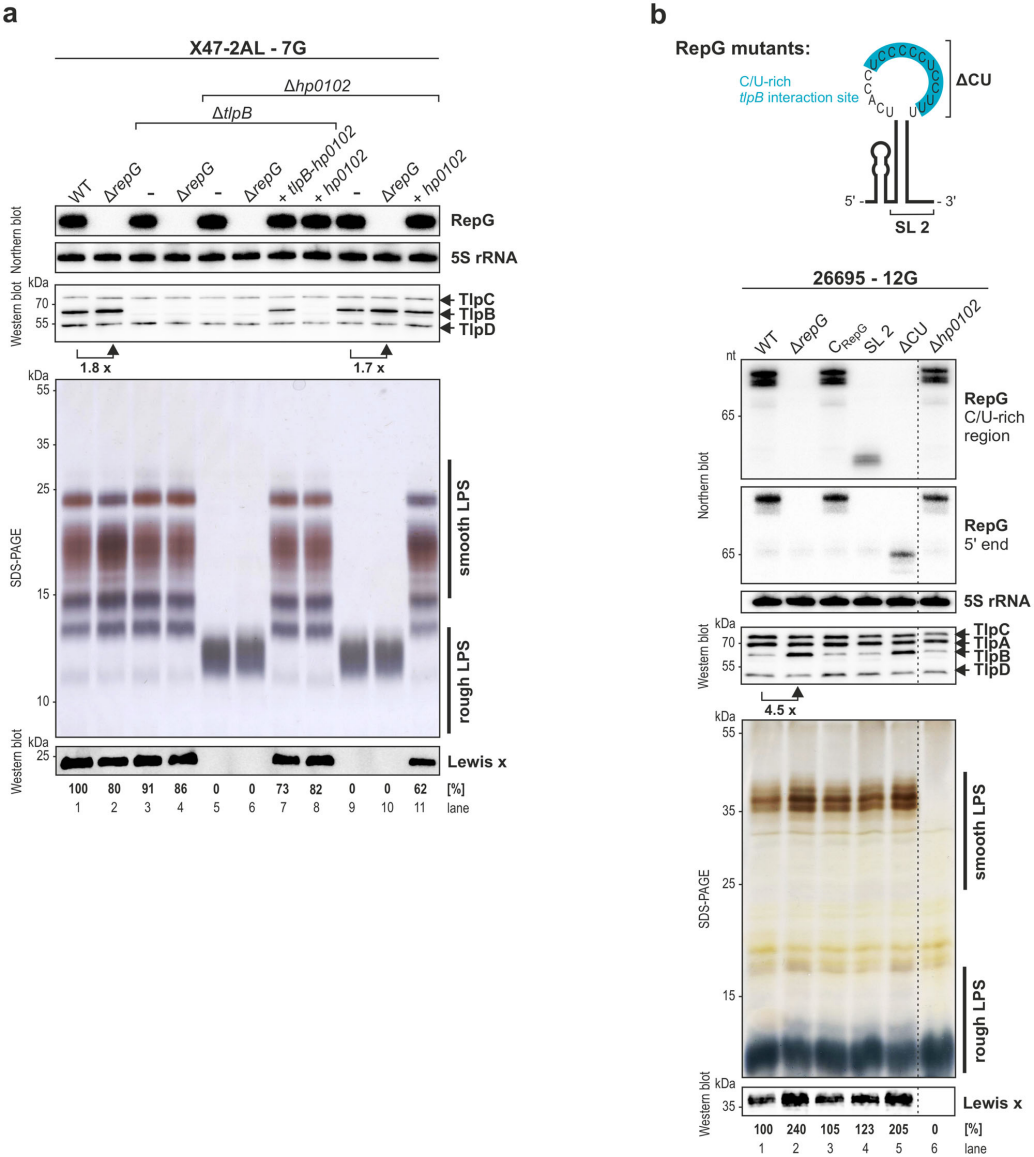
The *hp0102* gene is required for LPS O-antigen biosynthesis and is regulated by RepG sRNA. The WT and indicated mutant strains of *H. pylori* strains X47-2AL (**a**) and 26695 (**b**) were grown to exponential growth phase, and RNA and protein samples were analyzed by northern and western blot, respectively. **b** In 26695, the Δ*repG* mutant was complemented with wild-type RepG (C_RepG_) or mutant sRNAs. SL 2 consists of only the second stem-loop and in ΔCU the *tlpB* binding site (marked in blue) was replaced by an extra-stable tetraloop^21^. RepG sRNA was detected with CSO-0003 (C/U-rich terminator loop) and JVO-2134 (5’ end). 5S rRNA was used as loading control (JVO-0485). Expression of the chemotaxis receptors TlpA, TlpB, TlpC, and TlpD was analyzed using a polyclonal anti-TlpA22 antiserum. Please note that the Δ*hp0102* mutant was loaded/analyzed on the same northern and western blots like the WT and indicated sRNA mutant strains; however, samples were not loaded directly next to each other (indicated by dotted line). **a**, **b** LPS samples of the indicated strains were separated on 15% SDS-PAGE gels and either directly visualized by silver staining, or electro-blotted to PVDF membrane and probed with a Lewis x antigen-specific antibody. The results shown are representative of at least three independent experiments. Source data underlying (**a**, **b**) are provided as a Source data file.

The HP0102 protein sequence contains several motifs of the glycosyltransferase family 2 (GT-2; www.kegg.jp, http://pfam.sanger.ac.uk/, http://www.cazy.org/), suggesting HP0102 is involved in LPS biosynthesis. While at the time of this study, the precise function of HP0102 was unknown, it was recently described to act as a fucosyltransferase involved in O-chain biosynthesis in *H. pylori* strain G27^10^. In line with the study by Li and colleagues^10^, we also observed that *H. pylori* X47-2AL ∆*hp0102* deletion mutants have only rough-form LPS, i.e. lack the O-chains, and no Lewis x antigens could be detected on western blots (Fig. 4a, lanes 5–6 and 9–10). In contrast, the ∆*repG*, ∆*tlpB*, and ∆*tlpB*/∆*repG* mutant strains showed the same smooth LPS profiles (composed of lipid A-core and O-antigens) as wildtype, with comparable Lewis x antigens levels (Fig. 4a, lanes 1–4). Complementation of ∆*tlpB*-*hp0102* and ∆*hp0102* with either the *tlpB*-*hp0102* operon or with *hp0102* alone restored smooth LPS patterns and wild-type Lewis x expression (Fig. 4a, lanes 7–8 and 11). Next, we further validated this function of HP0102 in LPS biosynthesis in the most commonly utilized *H. pylori* laboratory strains, namely 26695 (Fig. 4b, lanes 1 and 6), J99, and G27 (Supplementary Fig. 5). Variations in overall LPS patterns were observed among different WT strains supporting previous reports on strain-specific LPS variations^8^. However, irrespective of the parental strain background, mutants lacking *hp0102* express only rough LPS. This confirms a conserved function of HP0102 essential for LPS O-chain biosynthesis and Lewis x antigen display, which is also in agreement with HP0102 homologs universally present in different *H. pylori* strains^10^. The LPS O-chains contribute to antigenicity and serospecificity of native LPS and play an important role in *H. pylori* virulence^11,31,32^. Accordingly, the complete abrogation of mouse colonization by the *H. pylori* X47-2AL *hp0102* mutant strain most probably results from its loss of the LPS O-chains.

In order to narrow down how HP0102 affects LPS O-chain biosynthesis, we constructed several additional LPS mutants (Supplementary Figs. 6 and 7a) in *H. pylori* strains 26695 and X47-2AL and compared their LPS profiles with the ∆*hp0102* mutants. These deletions affected LPS core oligosaccharide (HP1284^8^), various parts of the O-antigen (HP1039^33^; HP1581^33^; HP0159^34^ HP0826^35^), lipid A modulation (HP0579-0580^36^), or LPS O-chain translocation (HP1206^33^). Comparing their LPS profiles and Lewis x/y antigen expression to the patterns of the corresponding ∆*hp0102* mutants showed that the deep rough LPS phenotype of the HP0102 deletion mutant most closely resembled the pattern observed for the HP1039 and HP1581 mutant strains (Supplementary Fig. 7b, c). In addition, deletions of HP0102, HP1039, HP1581, HP0159, HP0826, and HP1206 resulted in the loss of both Lewis x and y antigens. These observations were consistent in both *H. pylori* strain backgrounds. These mutational analyses together with a previously missing enzyme for the conserved trisaccharide of the O-antigen (previously referred to as the outer core)^8^, indicated HP0102 is involved in the biosynthesis of the trio (Supplementary Fig. 7). Indeed, and in line with our observations, Li and colleagues revealed HP0102 as the fucosyltransferase of the LPS trisaccharide using mass spectrometry-based structural LPS analyses^10^. Because both, X47-2AL and 26695, showed stronger signals for Lewis x than Lewis y, all following experiments regarding LPS profiling and Lewis antigen expression were focused on western blot analyses of Lewis x.

### RepG regulates *tlpB-hp0102* and consequently LPS biosynthesis

To investigate whether RepG sRNA regulates expression of *hp0102* (in addition to *tlpB*) and in turn LPS biosynthesis, we examined LPS patterns and Lewis x antigen display in *H. pylori* strain 26695, in which *tlpB* is more strongly regulated by RepG than in strain X47-2AL (Supplementary Fig. 2). Deletion of *repG* resulted in increased band intensities in smooth LPS and Lewis x antigen levels in strain 26695 (Fig. 4b, lanes 1–2), indicating regulation of *hp0102* by RepG. Complementation of the Δ*repG* mutant with wild-type (C_RepG_) or a mutant RepG sRNA expressing only the second stem-loop with the C/U-rich *tlpB* binding site (SL2) restored wild-type LPS and Lewis x (Fig. 4b, lanes 3–4). In contrast, deletion of the previously identified C/U-rich *tlpB* interaction site in RepG (∆CU)^21^, led to increased O-antigen and Lewis x similar as observed for the *repG* deletion mutant (Fig. 4b, lane 5). These RepG mutant analyses demonstrate that the C/U-rich terminator loop of RepG is sufficient to repress both *tlpB* and *hp0102*.

### The G-repeat in the *tlpB-hp0102* leader determines LPS production

While RepG is highly conserved, the length of its G-repeat target in the *tlpB* leader varies among *H. pylori* isolates, resulting in strain-specific *tlpB* regulation^21^. RepG represses TlpB protein expression in *H. pylori* strains X47-2AL (7G, two-fold; Fig. 4a) and 26695 (12G, five-fold; Fig. 4b). In contrast, TlpB protein levels were unaffected or increased upon *repG* deletion in strains J99 (13G) and G27 (14G, two-fold), respectively (Supplementary Fig. 5). Deletion of *repG* did not significantly affect LPS profiles in strains X47-2AL, J99, and G27 (Fig. 4a, lane 2 and Supplementary Fig. 5), but resulted in increased band intensities in smooth LPS and Lewis x antigen levels in strain 26695 (Fig. 4b, lanes 1–2), confirming a strain-specific G-repeat-dependent *tlpB-hp0102* regulation.

**Fig. 5:**
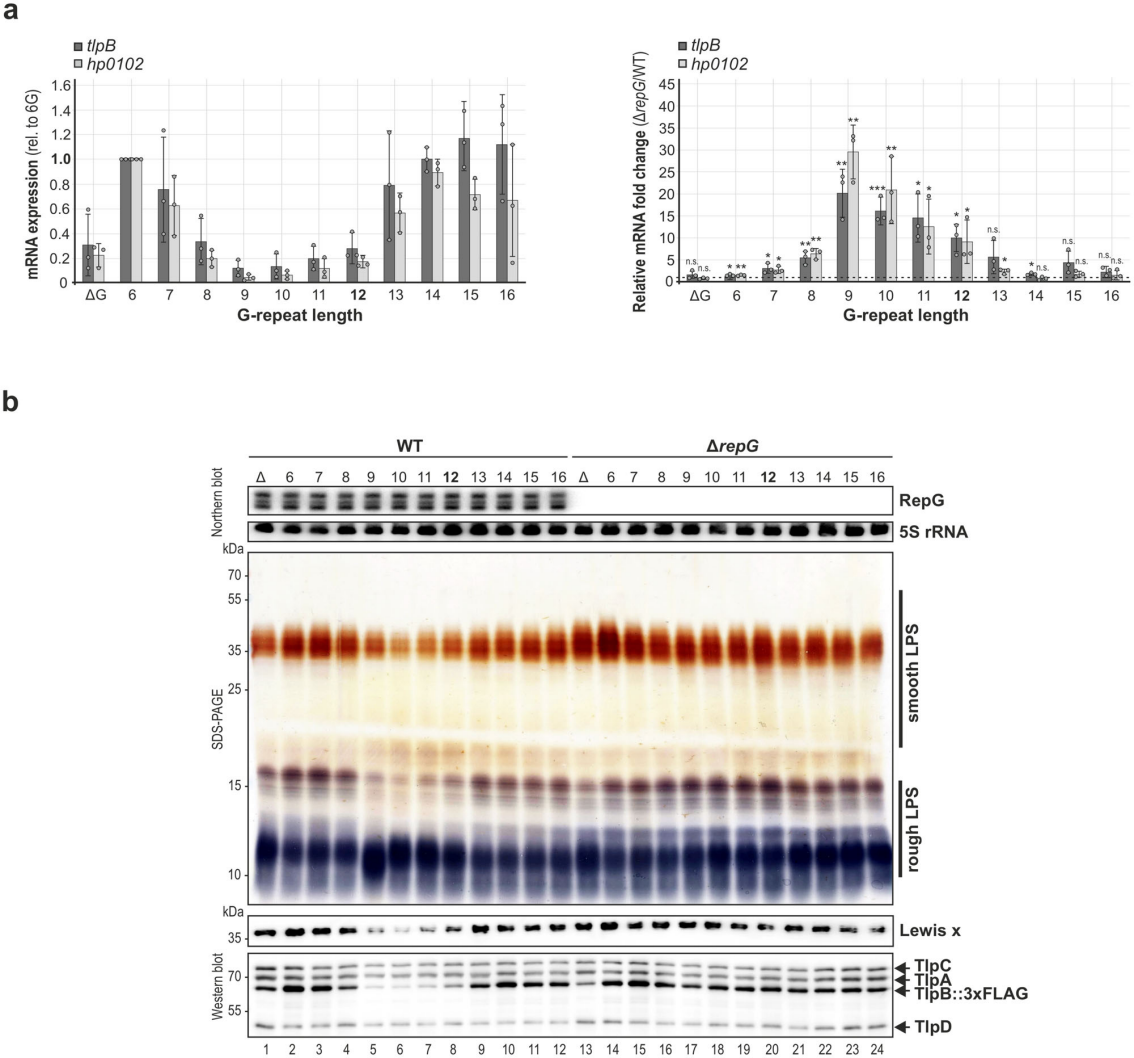
The G-repeat length impacts RepG-mediated *tlpB*-*hp0102* co-regulation and in turn smooth LPS production. **a** (Left panel) RT-qPCR of relative *tlpB* and *hp0102* mRNA levels of different *tlpB* leader mutants (ΔG, 6–16G) in the *H. pylori* 26695 wild-type background. The mRNA level of each gene in the *tlpB* 6G-leader mutant was used as reference and set to 1. (Right panel) Relative fold changes of *tlpB* and *hp0102* mRNA levels upon *repG* deletion in *H. pylori* 26695 *tlpB* leader mutants when compared to the respective wild-type backgrounds. The fold changes are shown as mean of three biological replicates with corresponding error bars (s.d.). ***—highly significant, *p*-value < 0.001; **—very significant, *p*-value < 0.01; *—significant, *p*-value < 0.05; n.s.—not significant; Student’s *t*-test, two-tailed. **b** The LPS patterns and Lewis x antigen levels of *tlpB* leader mutants in *H. pylori* 26695 wild-type and ∆*repG* mutant backgrounds were analyzed by silver staining and western blot analysis with anti-Lewis x antigen antibody, respectively. Expression of the chemotaxis receptors was analyzed by an anti-TlpA22 antiserum. Note that *tlpB* leader mutants express FLAG-tagged TlpB^21^. A representative silver-stained PAA-gel and western blot are shown (out of three independent experiments). Source data underlying (**a**, **b**) are provided as a Source data file.

To more systematically investigate whether the G-repeat length impacts RepG-mediated control of *hp0102*, the G-repeat was either deleted (ΔG) or mutated from 6 to 16 guanines (6–16G) in the *tlpB* mRNA leader of *H. pylori* 26695 expressing TlpB::3xFLAG (as in ref. ^21^). RT-qPCR analysis of *tlpB* and *hp0102* mRNA levels in the *tlpB* leader variants (∆G, 6–16G) of *H. pylori* 26695 revealed that expression of both genes is indeed dependent on the G-repeat length (Fig. 5a, left panel). While deletion of the G-repeat (ΔG) had only a minor influence on *tlpB* and *hp0102* mRNA levels when compared to wildtype (12G), expression of both genes was increased in the 6G-variant. A gradual decrease in *tlpB* and *hp0102* mRNA levels was observed with an increasing number of guanines in the *tlpB* mRNA leader, reaching a minimum level for 8–12Gs. Further extension of the G-stretch from 13–16Gs resulted again in elevated transcript abundances. Whereas deletion of *repG* affected neither *tlpB* nor *hp0102* expression in ∆G, 6G, and 13–16G, significantly increased mRNA levels were observed in the 7–12G leader mutants upon *repG* deletion (Fig. 5a, right panel).

Next, we investigated whether SSR-dependent regulation of the *tlpB-hp0102* operon also affects LPS biosynthesis. Every *tlpB* mRNA leader variant (∆G, 6–16G) of the wild-type background expresses smooth LPS. However, similar to gradual TlpB expression levels depending on the G-repeat length, varying O-chain and Lewis x profiles were detected (Fig. 5b, lanes 1–12). Whereas *tlpB* leader length variants of 6–8G and 13–16G displayed increased expression levels, decreased amounts of O-chains and Lewis x levels were detected for 9–11G variants in comparison to WT (12G; Fig. 5b and Supplementary Fig. 8). The gradual TlpB and LPS/Lewis x patterns were lost upon *repG* deletion, confirming that the G-repeat length indeed affects sRNA-mediated regulation. The strongest effects were observed in *tlpB* mRNA leader mutants that comprise a G-repeat of 9–12Gs, in which deletion of *repG* resulted in significantly increased O-chain and Lewis x levels. In contrast, LPS patterns remained almost unaltered in the ∆G, 6–8G, and 13–16G variants.

In conclusion, the amounts of Lewis x antigen and TlpB protein levels closely correlate in the varying G-repeat strains. RepG significantly represses TlpB protein and smooth LPS biosynthesis/O-antigen levels in *tlpB* leader variants that comprise a 9–12G-long repeat, in line with the previously defined optimal window for RepG-mediated repression^21^. Overall, these data show that the length of the G-repeat in the *tlpB* mRNA leader impacts RepG-mediated co-regulation of *tlpB* and *hp010*2 and consequently, smooth LPS production.

### Deletion of *hp0102* leads to reduced growth upon salt stress

**Fig. 6:**
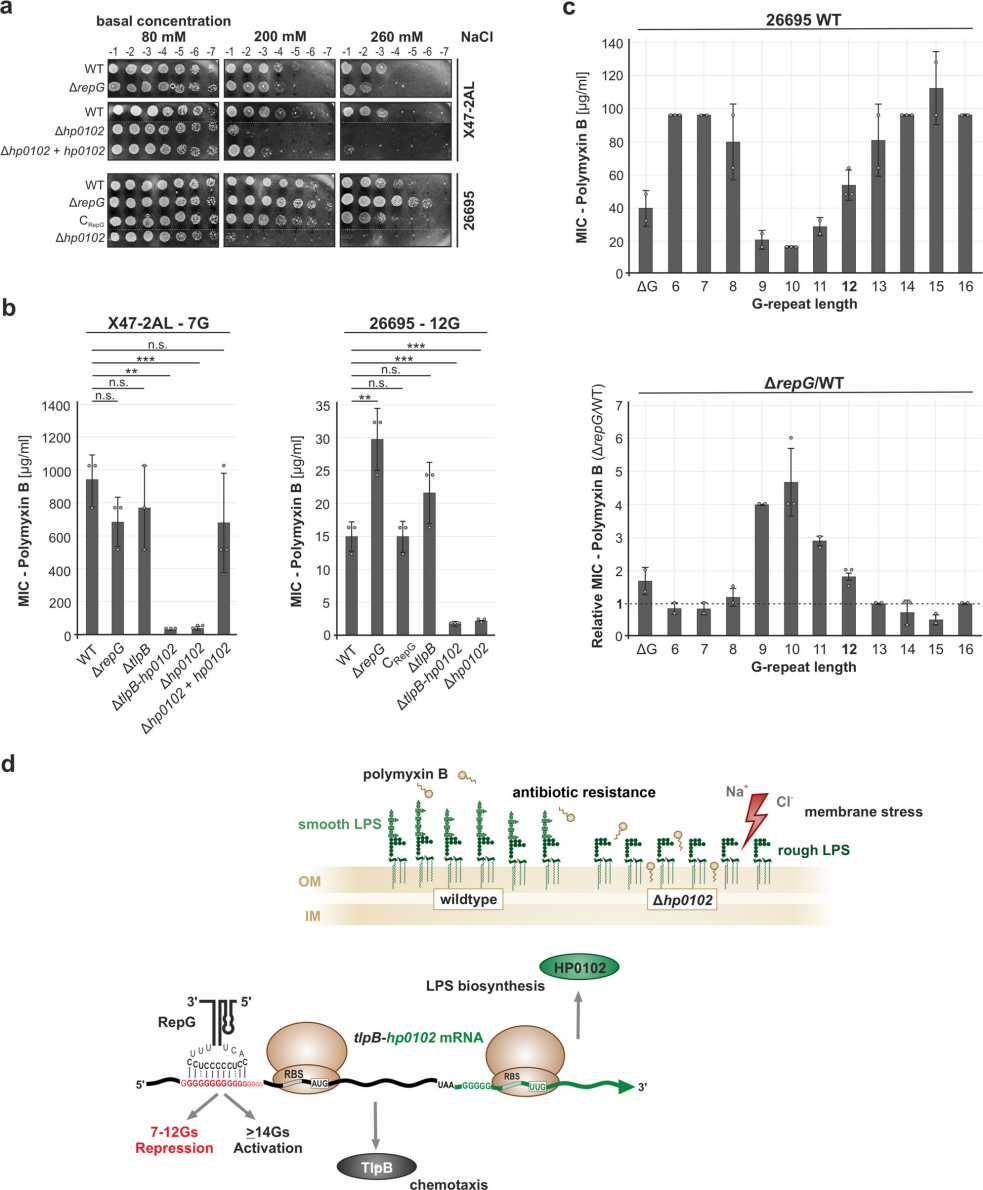
RepG affects sensitivity to salt stress and polymyxin B via regulation of *hp0102*. **a** *H. pylori* X47-2AL and 26695 WT and indicated mutant strains were grown in liquid culture to exponential growth phase (OD_600_ of 1). Indicated ten-fold dilutions of the bacterial suspensions were spotted on GC-agar plates containing 80 mM (basal concentration), 200 mM (mild stress), or 260 mM (harsh stress) sodium chloride. Plates were incubated for 3–5 days at 37 °C under microaerobic conditions. The results shown are representative of at least two independent experiments. **b** Polymyxin B sensitivity testing for *H. pylori* X47 and 26695 WT and indicated mutant strains using E-tests. Error bars indicate s.d. for *n* = 3 independent biological experiments. ****— highly significant, *p*-value < 0.0001; **—very significant, *p*-value < 0.01; n.s.—not significant; Student’s *t*-test, two-tailed. **c** (Upper panel) Polymyxin B MICs of *H. pylori* 26695 *tlpB* mRNA leader variants in the wild-type background. (Lower panel) Relative fold changes in polymyxin B sensitivity (MICs) upon *repG* deletion in different *tlpB* leader variants when compared to the respective wild-type backgrounds (*n* = 2 for WT/∆*repG*_∆G, ∆*repG*_6G, WT/∆*repG*_7-9G, WT/∆*repG*_11G, WT/∆*repG*_13G, WT/∆*repG*_15-16G; *n* = 3 for WT_6G, WT/∆*repG*_10G, WT/∆*repG*_12G, WT/∆*repG*_14G). **d** Model of RepG-mediated regulation of TlpB and HP0102. The genes encoding the chemotaxis receptor TlpB (dark gray) and the fucosyltransferase HP0102 (green) are transcribed as a bicistronic mRNA and play a role in chemotaxis^25^ and smooth LPS biosynthesis (light green) as well as protection against membrane stress (indicated by red arrow), respectively. Deletion of *hp0102* leads to loss of O-antigens and a rough LPS phenotype (dark green). Antisense base-pairing of the C/U-rich terminator loop of RepG sRNA to the homopolymeric G-repeat in the 5′ UTR of the *tlpB-hp0102* mRNA results in post-transcriptional co-regulation of *tlpB* and *hp0102* at the transcript and protein level. Depending on the G-repeat length, RepG mediates both repression (7–12Gs) and activation (≥14Gs) of *tlpB* and *hp0102*. OM/IM—outer/inner membrane, RBS—ribosome binding site, AUG/UUG—start codons, UAA—stop codon. Source data underlying (**b**, **c**) are provided as a Source data file.

LPS is essential for the integrity and functionality of the bacterial outer membrane and protects the bacteria against surface stress^37^. For example, divalent cation bridging of phosphates on lipid A and/or the core oligosaccharide of LPS contributes to the stabilization of the outer membrane and ensures its function as an effective permeability barrier. Bacteria with incomplete LPS, e.g., rough LPS, are more sensitive to environmental stresses, including osmotic stress^38^. To examine whether, or to which extent, the *hp0102*-dependent rough LPS phenotype affects *H. pylori* survival under osmotic stress, the growth of the wildtype and mutant strains of *H. pylori* X47-2AL, 26695, J99, and G27 was examined under elevated sodium chloride (NaCl) concentrations, an often-used method to simulate osmotic stress conditions. A drastic decrease in viable bacterial counts was observed on high-salt plates for all tested *H. pylori* mutants lacking *hp0102* (Fig. 6a and Supplementary Fig. 9a). Hence, HP0102-dependent smooth LPS production is required to maintain the integrity of the *H. pylori* cell envelope. Complementation of the *H. pylori* X47-2AL Δ*hp0102* mutant only partially restored wild-type growth (Fig. 6a), which might be due to a slightly different expression of HP0102 in the complementation strain. In line with *hp0102* being repressed by RepG in *H. pylori* strain 26695, deletion of *repG* rendered *H. pylori* more resistant to high-salt conditions (Fig. 6a). In contrast, an increased sensitivity toward osmotic stress was observed for the *H. pylori* G27 Δ*repG* mutant (Supplementary Fig. 9a). Because smooth LPS biosynthesis seems to be unaffected by RepG in G27 (Supplementary Fig. 5), this phenotype might be linked to the regulation of other, so far undefined, targets of RepG.

### RepG affects polymyxin B sensitivity by repression of *hp0102*

Modifications in the LPS structure have been shown to affect *H. pylori* resistance to cationic antimicrobial peptides (CAMPs^9,12^), which represent critical components of the human innate immune system. CAMPs interact with negatively charged phospholipid groups or the LPS lipid A anchor, inducing bacterial lysis and cell death. Using E-test strips, we measured the resistance of *H. pylori* X47-2AL, 26695, J99, and G27 wildtype and chosen mutant strains to polymyxin B (PxB), a membrane-targeting antibiotic and surrogate for host CAMPs. Irrespective of the parental strain background, Δ*hp0102* mutants displayed reduced minimal inhibitory concentrations (MICs) to PxB compared to WT (Fig. 6b and Supplementary Fig. 9b–d, Supplementary Table 1). Complementation of Δ*hp0102* in X47-2AL restored wild-type PxB susceptibility, suggesting that the alteration of the LPS structure through *hp0102* is associated with antibiotic sensitivity in *H. pylori*. While no significant effect was observed upon *repG* deletion in X47-2AL, J99, and G27, the 26695 Δ*repG* mutant was twice as resistant to PxB as the wildtype. Deletion of *tlpB* in X47-2AL leads to a negligible reduction in MIC and a non-significant increase in strain 26695. Thus, the observed effects on PxB sensitivity seem to be mediated primarily by modulation of *hp0102* expression (Fig. 6b, Supplementary Table 1). Moreover, deletion of *hp0102* affects PxB sensitivity more strongly compared to mutants defective in O-chain length (HP0826 and HP0159) but less pronounced when compared to deletion mutants completely devoid of O-chain (HP1039 and HP1581), the core oligosaccharide (HP1284), and lipid A (HP0579/80) (Supplementary Fig. 9c, d and Supplementary Table 1). This additionally supports the recently reported function of HP0102 as the LPS trisaccharide fucosyltransferase^10^.

Next, we characterized PxB sensitivity of *tlpB-hp0102* G-repeat leader variants of 26695 WT and Δ*repG*. While sensitivity to PxB was not affected in ΔG, the 6–8G and 13–16G variants were more resistant to PxB than the wildtype (12G; Fig. 6c, upper panel). Consistent with reduced smooth LPS expression (Fig. 5b), 9–11G mutants were more susceptible to PxB. In the ∆*repG* background, all *tlpB* mRNA leader variants displayed similar MICs (data not shown). Accordingly, deletion of *repG* resulted in two- to four-fold increased resistance to PxB for *tlpB* leader variants 9–12G (Fig. 6c, lower panel), whereas MICs remained unaltered in almost all other leader mutants. Taken together, these data demonstrate that RepG-mediated expression control of *hp0102* leads to gradual control of LPS O-chain synthesis and thereby contributes to the integrity and permeability of the bacterial membrane and *H. pylori* susceptibility to PxB.

## Discussion

Here, we showed that the *H. pylori* HP0102 protein is involved in LPS O-chain production, essential for murine stomach colonization and important for resistance to the membrane-targeting antibiotic PxB. Moreover, we demonstrated that *hp0102* is post-transcriptionally regulated by RepG sRNA through base-pairing to a variable, homopolymeric G-repeat in the 5′ UTR of the bicistronic *tlpB-hp0102* mRNA. In contrast to SSR-based phase-variable ON/OFF gene expression switches, this unique mode of regulation allows for a fine-tuned control of *hp0102* and in turn smooth LPS biosynthesis, which might be required for *H. pylori* adaptation to different host niches.

Binding of RepG to the G-repeat upstream of the *tlpB* open reading frame is sufficient to regulate *tlpB* and *hp0102* both at the transcript and protein level (Fig. 2). Our GFP reporter assay showed that *hp0102* translation is likely independent of *tlpB*, suggesting that RepG-mediated repression of *hp0102* is rather based on ribosomal recruitment and/or destabilization of the entire mRNA than on direct translational coupling. RepG regulates *tlpB* expression at the translational level and induces structural rearrangements within the *tlpB* mRNA when binding to the G-repeat^21^. Sequestration of the G-repeat, which is located relatively far upstream of the RBS and might function as a translational enhancer and/or ribosome standby site, could affect both *tlpB* and *hp0102* translation similarly as previously shown for other sRNAs^39,40^. Furthermore, structural rearrangements caused by RepG binding might also lead to the inhibition of translation elongation and/or whole transcript destabilization by RNase recruitment^41^.

Bacterial adaptive changes, including modulation of LPS synthesis and structure, play an important role during infection. In our study, we observed *H. pylori* Δ*hp0102* mutants display rough LPS phenotypes, indicative of O-antigen loss, supporting its recent identification as the trisaccharide fucosyltransferase^10^. *H. pylori* LPS can mimic host structures and is essential for resistance to host-derived CAMPs^6^, making it a key surface determinant required for colonization and persistence as shown in rodent models^12,31,32^. In line with a previous transposon mutagenesis screen in strain G27, which indicated *hp0102* is a candidate gene required for colonization in mice^42^, we observed that mice stomach colonization of an *H. pylori* X47-2AL ∆*hp0102* mutant is completely abolished. Similarly, other *H. pylori* glycosyltransferases involved in LPS biosynthesis are also essential for colonization of mice^8,43^. As LPS core and variable Lewis antigens facilitate immune escape^11,44^ and promote adherence to the gastric epithelium^45–47^, the Δ*hp0102* colonization defect might be associated with an enhanced susceptibility of this mutant to the inflammatory host response and membrane stress within the host and/or its impaired ability to bind to the gastric epithelium. Other functions might also be affected in the *H. pylori* ∆*hp0102* mutant, such as a recently reported modulation of the expression of the major virulence factor CagA as well as *H. pylori* chemotaxis^48^.

While chemotaxis in *H. pylori* is important for proper bacterial orientation and localization in the gastric glands^25,49^, the X47-2AL ∆*tlpB* mutant presented only a mildly reduced murine stomach colonization (Fig. 3) and no defect during competitive co-infection experiments^26,27^. Together with our data, this indicates that the TlpB chemotaxis receptor is not necessarily required for bacterial growth in the rodent stomach. Further studies are required to evaluate the importance of the *tlpB-hp0102* operon co-regulation and its functional implications for *H. pylori* persistence.

Diverse pathogens benefit from SSR-mediated phase variation of surface structures as these allow them to rapidly adapt to changes in their host environment^50,51^. The *H. pylori* genome contains variable SSRs in a number of outer membrane proteins, adhesins, or LPS-biosynthetic or -modifying enzymes (Supplementary Table 2)^52,53^. When sequentially isolated from human patients^54–58^ or re-isolated from animal colonization experiments^59,60^, even *H. pylori* strains within the same host exhibited varying G-repeat length in the *tlpB* 5′ UTR (Supplementary Table 3). This suggests that *tlpB-hp0102* is associated with a phase-variable SSR. So far, Lewis antigen-producing fucosyltransferases have been described to be controlled by SSRs in an ON/OFF manner^14–16,60–62^. In contrast, we uncovered a gradual regulation of an operon encoding two proteins important for *H. pylori* colonization, namely the chemotaxis receptor TlpB and HP0102 required for O-chain display, by a sRNA that targets a SSR in the mRNA leader sequence, resulting in gradual changes of smooth LPS. This “LPS fine-tuning” might help *H. pylori* to balance the fine line between host immune evasion and lack of sufficient adherence potential^63–65^. The here described gradual display of LPS O-chain might increase heterogeneity in the population, enabling *H. pylori* to adapt and survive in the gastric mucosa of individual host niches and thus contributing to persistence. HP0102-mediated structural LPS changes can support this adaptation as demonstrated by an increased sensitivity of a *hp0102* mutant to high salt concentrations and to membrane-targeting antibiotics such as PxB, which is a surrogate of host-derived CAMPs. The regulation of *hp0102* by the RepG sRNA thereby adds to a number of bacterial sRNAs that impact antibiotic resistance, e.g., by regulating expression of drug transporters or efflux proteins, biofilm formation, or metabolic enzymes involved in cell envelope synthesis or LPS biosynthesis^66^.

The RepG-dependent regulatory mechanism presents several unique features. We demonstrated that the G-repeat length mediates repression (7–12Gs) or activation (≥14Gs) of the *tlpB*-*hp0102* operon by RepG. This is a unique regulatory mechanism linking post-transcriptional gene expression control to phase variation. To the best of our knowledge, intergenic SSRs that affect full-length LPS O-chain display in a reversible and rheostat-like fashion at the post-transcriptional level, as described in this study, have not been identified in other bacterial pathogens so far. Besides *hp0102*, three other LPS-modifying enzymes are associated with intergenic SSRs in *H. pylori* strain 26695 (Supplementary Table 2). Whether these intergenic SSRs affect promoter strength and/or are targeting sites for sRNAs still needs to be examined. At the transcriptional level, for example, length variation of a promoter-associated, intergenic SSR has been described to result in low, mediate, and high expression levels of the major *H. pylori* adhesin SabA^67^.

Future experiments are needed to evaluate if the presence or expression of RepG sRNA itself might provide a selective force toward a certain length of the G-repeat, thereby influencing the rate of SSR polymorphism. Phase variation is usually stochastic and random; however, gene regulation has been shown to be integrated with phase variation events. For example, phase variation of methyltransferases can modulate bacterial gene expression via epigenetic mechanisms^68^ and Dam methylation can impact phase-variable ON/OFF switching^69^. Moreover, sRNA-mediated regulation of the P-fimbriae phase regulator *papI* affects the ON/OFF switch and, thus, expression of P-fimbriae on the surface of uropathogenic *E. coli*^70^. And transcription of a *cis*-encoded sRNA antisense to the *pilE* promoter impacts pilin antigenic variation in *Neisseria meningitidis*^71^. Recently, we and others also uncovered that sRNAs themselves can be associated with SSRs and identified a phase-variable sRNA, NikS, that acts as a global post-transcriptional regulator of the major virulence genes of *H. pylori*^72,73^.

In *H. pylori*, alterations in the LPS structure of *H. pylori* have been described at low pH^74^. The post-transcriptional regulation of the *tlpB-hp0102* operon through RepG might provide an additional layer of LPS gene expression control under varying environmental conditions. As antisense transcription has been identified to various genes involved in *H. pylori* LPS biosynthesis^28^, regulation of surface structures by *cis-* or *trans*-encoded antisense RNAs might be a more general theme in *H. pylori* gene expression control. In conclusion, our study has unraveled a rheostat-like type of phase variation-dependent, post-transcriptional regulation of surface structures that are important for the colonization capacity of a bacterial pathogen.

## Methods

### Bacterial strains, oligonucleotides, and plasmids

*Helicobacter pylori* and *Escherichia coli* strains are listed in Supplementary Table 4. DNA oligonucleotides and plasmids are summarized in Supplementary Tables 5 and 6, respectively. Sequence information on 5′ UTRs or coding sequences of *tlpB* and *hp0102* used in the GFP-reporter assays and *tlpB* leader variants are shown in Supplementary Tables 7 and 8, respectively.

### Bacterial growth

*E. coli* strains were grown in Luria Bertani (LB) medium supplemented with 100 µg/ml ampicillin, 20 µg/ml chloramphenicol, 20 µg/ml kanamycin, and/or 10 µg/ml gentamicin if applicable. *H. pylori* strains were grown on GC-agar (Oxoid) plates supplemented with 10% horse serum (DHS, Biochrom AG), 1% vitamin mix, 10 µg/ml vancomycin, 5 µg/ml trimethoprim, and 1 µg/ml nystatin. For transformant selection and growth of mutant strains, 20 µg/ml kanamycin, 20 µg/ml chloramphenicol, 10 µg/ml gentamicin or 10 µg/ml erythromycin were added. For liquid cultures, 15 or 50 ml Brain Heart Infusion medium (BHI, Roth) supplemented with 10% FBS (Biochrom AG) and 10 µg/ml vancomycin, 5 µg/ml trimethoprim, and 1 µg/ml nystatin were inoculated with *H. pylori* strains from plates to a final OD_600_ of 0.02–0.04 and grown under agitation at 140 rpm in 25 cm^3^ or 75 cm^3^ cell culture flasks. Bacteria were grown at 37 °C in a HERAcell 150i incubator (Thermo Scientific) in a microaerobic environment (10% CO_2_, 5% O_2_, and 85% N_2_).

### Construction of *Helicobacter pylori* mutant strains

All mutant strains are listed in Supplementary Table 4. Mutants were constructed by homologous recombination and natural transformation of PCR-amplified constructs carrying either the *aphA-*3 kanamycin^75^, the *catGC* chloramphenicol^76^, the *aac*(3)-IV apramycin/gentamicin^77^ or *rpsL-erm* erythromycin^78^ resistance cassette flanked by ~500 bp homology regions up- and downstream of the respective genomic locus. Briefly, *H. pylori* was grown from frozen stocks, passaged twice, then streaked on a fresh GC-agar plate and grown for 6–8 h at 37 °C under microaerobic conditions. For transformation, 500 ng up to 1 µg purified PCR product was added to the cells. After incubation for 14–16 h at 37 °C, cells were re-streaked on selective plates with the corresponding antibiotic. Genomic DNA (gDNA) of mutants was isolated using NucleoSpin Plasmid Kit according to the manufacturer’s instructions and mutants were checked by PCR and sequencing.

The *H. pylori* 26695 sRNA mutant (Δ*repG*, C_RepG_, SL 2, ΔCU) as well as Δ*tlpB* deletion strains, and *tlpB* mRNA leader variants (ΔG, 6–16G in wild-type and Δ*repG* mutant strain backgrounds), and the *repG* deletion mutants in diverse *H. pylori* strains (G27 and J99) were constructed in our previous study^21^.

### Construction of X47-2AL mutant strains for mouse infections

To delete *repG* in the mouse-adapted *H. pylori* strain X47-2AL, a Δ*repG* deletion construct (*aphA*-3 flanked by 500 nt up- and downstream of *repG*) was amplified by PCR using JVO-5070/-5072 and gDNA from JVS-7014 (*H. pylori* 26695 Δ*repG*, ref. ^28^). The purified PCR product was transformed into CSS-0996 (*H. pylori* X47-2AL wildtype). Deletion of *repG* was verified by PCR using JVO-5069/-5257 on gDNA, resulting in strain CSS-0997 (X47-2AL Δ*repG*).

The *tlpB* gene was deleted from strain CSS-0996 (*H. pylori* X47-2AL wildtype) by insertion of the *aac*(3)-IV cassette. To avoid potential polar effects of Δ*tlpB* on the expression of the downstream gene *hp0102*, the *tlpB* coding region was replaced by a non-polar gentamicin resistance cassette (*aac*(3)-IV), leaving the *tlpB* promoter and 5′ UTR intact. Therefore, a plasmid containing the *aac*(3)-IV cassette flanked by 500 nt up- and downstream of the *H. pylori* strain X47-2AL *tlpB* locus was cloned into *E. coli*. 500 nt up- and downstream of the *tlpB* open reading frame were amplified from gDNA of strain CSS-0996 (*H. pylori* X47-2AL wildtype) using CSO-0039/-0040. The resulting PCR product was *Xba*I/*Xho*I digested and introduced into likewise digested pJV752-1. Next, the resulting plasmid (pBA5-4) was used as a template for a PCR with CSO-0942/-1745 to replace the *aac*(3)-IV cassette with the *tlpB* open reading frame using an *Eco*RI restriction site. In parallel, a PCR with phosphorylated CSO-0263 and CSO-0293 on pUC1813apra^77^ was performed and the resulting PCR product was digested with *Eco*RI. Both *Eco*RI*-*digested PCR products (plasmid with *tlpB* up- and downstream region as well as *aac*(3)-IV) were ligated and transformed into *E. coli*. Insertion of the *aac*(3)-IV cassette was verified by colony PCR (pZE-A/CSO-0293), resulting in pBA13-5. A PCR product amplified with CSO-0039/-0040 from pSP13-5 was transformed into *H. pylori* X47-2AL (CSS-0996). Positive gentamicin-resistant mutants were confirmed by PCR on gDNA using CSO-0051/-0293, resulting in strain CSS-1123 (X47-2AL Δ*tlpB*).

An analogous cloning strategy was used for the construction of the *tlpB*-*hp0102* double deletion mutant (Δ*tlpB*-*hp0102*) and deletion of *hp0102* alone (Δ*hp0102*). Please note that in these mutants, the *aac*(3)-IV cassette was inserted 77 nt upstream of the *hp0102* stop codon to avoid interference with *hp0101* expression. For deletion of the entire *tlpB*-*hp0102* operon, a PCR-fragment containing 500 nt downstream of the *H. pylori* X47-2AL *hp0102* open reading frame (CSO-0871/-1359 on gDNA of CSS-0996) was inserted into pBA13-5 (CSO-0874/0293 on pBA13-5) using oligo-introduced *Eco*RI/*Xho*I restriction sites. The ligated PCR-products were transformed into *E. coli*, resulting in pSP127-3. Insertion of the 500 nt downstream of *hp0102* was verified by colony PCR using pZE-A/CSO-1359. The *tlpB*(500up)-*aac*(3)-IV-*hp0102*(500down) deletion construct was amplified from pSP127-3 with CSO-0040/-1359 and transformed into *H. pylori* X47-2AL (CSS-0996). Positive gentamicin-resistant mutants were confirmed by PCR on gDNA using CSO-0051/-0293, resulting in strain CSS-1743 (X47-2AL Δ*tlpB*-*hp0102*).

In order to delete *hp0102* alone, a PCR-product containing 500 nt upstream of the *hp0102* open reading frame was amplified using CSO-1737/-1739 on gDNA of CSS-0996. This PCR product was *Xba*I*/Bam*HI digested and ligated together with a likewise digested PCR-product amplified from pSP127-3 using CSO-0873/3195. The resulting plasmid pSP186-2 was checked by PCR using pZE-A and CSO-1739. A PCR product (*hp0102*(500up)-*aac*(3)-IV-*hp0102*(500down)) amplified from pSP186-2 with CSO-1737/-1359 was transformed into *H. pylori* X47-2AL wildtype (CSS-0996). Positive gentamicin-resistant mutants were confirmed by PCR on gDNA (CSO-1738/0293), resulting in CSS-2019 (X47-2AL Δ*hp0102*).

For construction of double deletion mutants Δ*tlpB*/Δ*repG*, Δ*tlpB*-*hp0102*/Δ*repG,* and Δ*hp0102*/Δ*repG*, the sRNA deletion construct (*aphA*-3 flanked by 500 nt up- and downstream of *repG*) amplified by PCR using JVO-5070/-5072 on gDNA from JVS-7014 (*H. pylori* 26695 Δ*repG*) was transformed into CSS-1123 (X47-2AL Δ*tlpB*), CSS-1743 (X47-2AL Δ*tlpB*-*hp0102*), and CSS-2019 (X47-2AL Δ*hp0102*). The double deletion strains CSS-1769 (X47-2AL Δ*tlpB*/Δ*repG*), CSS-1773 (X47-2AL Δ*tlpB*-*hp0102*/Δ*repG*) and CSS-2022 (X47-2AL Δ*hp0102*/Δ*repG*) were verified by PCR using JVO-5069/-5257.

The Δ*tlpB*-*hp0102* and Δ*hp0102* deletion mutants were complemented with either the entire *tlpB*-*hp0102* operon or *hp0102* alone in the unrelated *rdxA* locus. Therefore, plasmids containing *rdxA*(500up)-*aphA*-3-*tlpB*-*hp0102*-*rdxA*(500down) or *rdxA*(500up)-*aphA*-3-P_*tlpB*_*hp010*2-*rdxA*(500down) were constructed in *E. coli*. First, *Cla*I/*Nde*I-digested PCR products amplified with CSO-1740/-1741 on gDNA of CSS-0996 (*H. pylori* X47-2AL) and CSO-0146/-0147 on pBA4-2 (pSP39-3 carrying *aac*(3)-IV gentamicin resistance cassette) were ligated and transformed into *E. coli*, resulting in pSP189-4. The *aac*(3)-IV gentamicin resistance cassette from pSP189-4 was exchanged with the *aphA*-3 kanamycin resistance cassette by ligation of *Bam*HI/*Nhe*I*-*digested PCR products amplified with CSO-0940/-0941 on pSP189-4 and CSO-1813/-1814 on gDNA of JVS-7014 (*H. pylori* 26695 Δ*repG*). The resulting plasmid pSP190-1 (*rdxA*(500up)-*aphA*-3-*tlpB*-*hp0102*-*rdxA*(500down)) was used as template for cycle-PCR using CSO-1743/-1742. This PCR product was *Dpn*I-digested and directly transformed into *E. coli*, resulting in pSP192-1 (*rdxA*(500up)-*aphA*-3-P_*tlpB*_*hp0102*-*rdxA*(500down)). PCR products amplified by CSO-0017/-0018 on pSP190-1 and pSP192-1 were used for transformation in CSS-1743 (X47-2AL Δ*tlpB*-*hp0102*) and CSS-2019 (X47-2AL Δ*hp0102*). The obtained, kanamycin-resistant strains CSS-2046 (X47-2AL Δ*tlpB*-*hp0102* + *tlpB*-*hp0102*), CSS-2080 (X47-2AL Δ*tlpB*-*hp0102* + *hp0102*), and CSS-2087 (X47-2AL Δ*hp0102* + *hp0102*) were verified by PCR using oligos CSO-0207/-1813 and sequencing by CSO-0206/-0086.

### Construction of Δ*hp0102* in *H. pylori* 26695, G27, and J99

The *hp0102* gene was deleted from strain CSS-0004 (*H. pylori* 26695 wildtype), CSS-0010 (*H. pylori* G27 wildtype) and CSS-0001 (*H. pylori* J99 wildtype) by insertion of the *rpsL-erm* cassette, which confers dominant streptomycin susceptibility and erythromycin resistance^78^. A plasmid containing the *rpsL-erm* cassette flanked by 500 nt up- and downstream of the *hp0102* open reading frame was generated in *E. coli*. A PCR product corresponding to 500 nt upstream of *hp0102* (CSO-0869/-0870 on gDNA of CSS-0004) was *Bam*HI/*Xho*I-digested and ligated into likewise digested pSP60-2^21^, resulting on pBA1-1. About 500 nt downstream of *hp0102* were introduced into pBA1-1 by ligation of *Eco*RI/*Xba*I-digested PCR products amplified by CSO-0871/-0872 on gDNA of CSS-0004 and CSO-0309/-0873 on pBA1-1. The resulting plasmid pBA7-4 was used for PCR with oligonucleotides CSO-0870/-0872 and the obtained PCR product was transformed into CSS-0004, CSS-0010, and CSS-0001. Positive erythromycin-resistant clones were checked by PCR on gDNA using CSO-0051 and CSONIH-0033, resulting in strain CSS-1000 (26695 Δ*hp0102*), CSS-1007 (G27 Δ*hp0102*), and CSS-1019 (J99 Δ*hp0102*).

### Construction of *H. pylori* LPS mutants in 26695 and X47-2AL

Deletion of genes involved in various steps of LPS biosynthesis in *H. pylori* strains 26695 and X47-2AL were constructed by overlap PCR and subsequent double-crossover homologous recombination. In addition, for strain 26695, new deletion mutants for *tlpB*, *tlpB-hp0102*, and *hp0102* carrying the *aphA-*3 instead of the *rpsL-erm* cassette were constructed. Overlap PCR products carried resistance cassettes flanked by ~500 nt of homologous sequence up- and downstream of the gene to be deleted. Resistance cassettes used for cloning were either *aphA-*3 (kanamycin) for strain 26695 or *aac*(3)-IV (apramycin/gentamicin) for X47-2AL. Non-polar resistance cassettes were amplified with HPK1/HPK2 from gDNA of *H. pylori* strain 26695 C_RepG_ (CSS-0046)^21^, or with CSO-SP008 and CSO-SP009 from pUC1813apra^77^.

As an example, deletion of *hp1284* in *H. pylori* strain 26695 will be described in detail. About 500 nt up- and downstream of the *hp1284* coding region were amplified using CSO-SP010 × CSO-SP066 and CSO-SP067 × CSO-SP013, respectively. The antisense oligonucleotide of the *hp1284* upstream region (CSO-SP066) and the sense oligonucleotide of the *hp1284* downstream region (CSO-SP013) contained 21 and 25 nt overlap, respectively, with the sense and antisense oligonucleotide used to amplify the *aphA-*3 resistance cassette (HPK1 × HPK2). The final overlap PCR product was amplified with CSO-SP010 and CSO-SP013 using a 1:1:2 ratio of up-/downstream region of *hp1284* and *aphA-*3 cassette. The program for the overlap PCR was as follows: 1 cycle of [98 °C, 1 min; 61 °C, 1 min; 72 °C, 10 min; 98 °C, 1 min], 40 cycles of [98 °C, 15 s; 57 °C, 30 s; 72 °C, 1 min], followed by 72 °C for 10 min. After size confirmation via agarose gel electrophoresis, the purified PCR product (Macherey-Nagel NucleoSpin PCR cleanup kit) was naturally transformed into the recipient *H. pylori* 26695 wild-type background (CSS-0004). Kanamycin-resistant clones were verified via colony PCR with CSO-SP014 and HPK2 resulting in the final *hp1284* deletion mutant (CSS-5928; ∆*hp1284*). Deletion mutants for *tlpB*::*aphA-*3 (CSS-5924; ∆*tlpB* (Kan)), *tlpB-hp0102*::*aphA-*3 (CSS-5926; ∆*tlpB-hp0102*), *hp0102*::*aphA-*3 (CSS-5942; ∆*hp0102* (Kan)), *hp1039*::*aphA-*3 (CSS-5930; ∆*hp1039*), *hp1581*::*aphA-*3 (CSS-5932; ∆*hp1581*), *hp0159*::*aphA-*3 (CSS-5934; ∆*hp0159*), *hp0826*::*aphA-*3 (CSS-5936; ∆*hp0826*), *hp0579-0580*::*aphA-*3 (CSS-5938; ∆*hp0579-580*), *hp1206*::*aphA-*3 (CSS-5940; ∆*hp1206*) in *H. pylori* strain 26695, and *hp1284*::*aac*(3)-IV (CSS-5910; X47-2AL ∆*hp1284*), *hp1039*::*aac*(3)-IV (CSS-5912; X47-2AL ∆*hp1039*), *hp1581*::*aac*(3)-IV (CSS-5914; X47-2AL ∆*hp1581*), *hp0159*::*aac*(3)-IV (CSS-5916; X47-2AL ∆*hp0159*), *hp0826*::*aac*(3)-IV (CSS-5918; X47-2AL ∆*hp0826*), *hp0579-0580*::*aac*(3)-IV (CSS-5920; X47-2AL ∆*hp0579-580*), *hp1206*::*aac*(3)-IV (CSS-5922; X47-2AL ∆*hp1206*) in *H. pylori* strain X47-2AL were constructed analogously.

### Cloning of translational reporter fusions to *gfpmut3*

For the generation of *hp0102* translational reporter fusions, the N-terminal coding region of *hp0102* was fused to *gfpmut3* and introduced together with the *catGC* resistance cassette^76^ into the *rdxA* locus of *H. pylori* strain G27. The first ten amino acids of the *hp0102* coding region together with the upstream-encoded gene *tlpB*, including the *tlpB* promoter and 5′ UTR (26695, 12G), were amplified from gDNA of *H. pylori* 26695 (CSS-0004) using oligos CSO-0581/-1803. The purified PCR product was digested with *Cla*I*/Nhe*I and ligated with a likewise digested PCR product, which was amplified from pMA5-2 (*cagA* 28^th^::*gfpmut3*) using CSO-0146/-0683, resulting in pSP195-6 (*tlpB*-*hp0102* 10^th^::*gfpmut3*). Additional *hp0102* 10^th^::*gfpmut3* reporter fusions were generated by cycle-PCR on pSP195-6 with CSO-1984/-1985 (*tlpB*_mini_-*hp0102*), CSO-2055/-2056 (P_*tlpB*_*hp0102*), CSO-2052/-2053 (*tlpB*-*hp0102* ΔG^*hp0102*^), CSO-2053/-2061 (*tlpB*-*hp0102* ATTTA^*hp0102*^) and CSO-2274/-2275 (*tlpB*_stop_-*hp0102*), respectively. The obtained PCR products were *Dpn*I*-*digested, self-ligated, and transformed into *E. coli*. Positive clones were selected on plates with 100 μg/ml ampicillin and 20 µg/ml chloramphenicol, and confirmed by colony PCR using oligos pZE-*Xba*I/CSO-0581. The resulting plasmids pSP197-3 (*tlpB*_mini_-*hp0102*), pSP198-4 (P_*tlpB*_*hp0102*), pSP200-2 (*tlpB*-*hp0102* ΔG^*hp0102*^), pSP201-1 (*tlpB*-*hp0102* ATTTA^*hp0102*^), and pSP205-17 (*tlpB*_stop_-*hp0102*) were validated by sequencing with CSO-0206/JVO-0155. Afterwards, these plasmids were used as templates for PCR with CSO-0017/-0018. The PCR products with different *hp0102*::*gfpmut3* reporter fusions were transformed into *H. pylori* G27 wildtype (CSS-0010) and/or Δ*repG* (CSS-0169). Positive transformants were checked by PCR with CSO-0205/-0207 and the in-frame fusion of *hp0102* to *gfpmut3*, as well as *tlpB*, was verified by sequencing with CSO-0206/JVO-0155. The corresponding *H. pylori* G27 strains are CSS-2104 (*tlpB*-*hp0102* 10^th^::*gfpmut3*), CSS-2107 (*tlpB*-*hp0102* 10^th^::*gfpmut3*/Δ*repG*), CSS-2116 (*tlpB*_mini_-*hp0102* 10^th^::*gfpmut3*), CSS-2119 (*tlpB*_mini_-*hp0102* 10^th^::*gfpmut3*/Δ*repG*), CSS-2138 (P_*tlpB*_*hp0102* 10^th^::*gfpmut3*), CSS-2141 (P_*tlpB*_*hp0102* 10^th^::*gfpmut3*/Δ*repG*), CSS-2150 (*tlpB*-*hp0102* ΔG^*hp0102*^ 10^th^::*gfpmut3*), CSS-2153 (*tlpB*-*hp0102* ΔG^*hp0102*^ 10^th^::*gfpmut3*/Δ*repG*), CSS-2156 (*tlpB*-*hp0102* ATTTA^*hp0102*^ 10^th^::*gfpmut3*), CSS-2159 (*tlpB*-*hp0102* ATTTA^*hp0102*^ 10^th^::*gfpmut3*/Δ*repG*), CSS-3241 (*tlpB*_stop_-*hp0102* 10^th^::*gfpmut3*) and CSS-3244 (*tlpB*_stop_-*hp0102* 10^th^::*gfpmut3*/Δ*repG*).

### RNA preparation, northern blot, and RT-qPCR

If not mentioned otherwise, total RNA from *H. pylori* grown in liquid culture to mid-exponential growth phase (OD_600 nm_ of ~0.7–1) was extracted using the hot phenol method as described previously^21^. For northern blot analysis, 5–10 µg total RNA was separated on 6% polyacrylamide (PAA) gels containing 7 M urea and blotted to Hybond-XL membranes (GE-Healthcare). After blotting, total RNA was UV cross-linked to the membrane and hybridized with 5′ end labeled (γ^32^P) DNA oligonucleotides as described previously^21^.

For RT-qPCR analysis, RNA samples were digested with DNaseI (Fermentas) to remove genomic DNA. All RT-qPCR experiments were carried out in at least technical and biological triplicates on a CFX96 system (Biorad) using Power SYBR Green RNA-to-C_T_^TM^ 1-Step kit (Applied Biosystems by Thermo Fisher Scientific) according to the manufacturer’s instructions. Fold-changes were determined by the 2^(−ΔΔCT)^-method^79^. The specific primer sets are JVO-5267/-5268 for *tlpB* mRNA, CSO-0867/-0868 for *hp0102* mRNA, and CSO-1173/-1174 for 6S rRNA, which served as internal standard.

### RT-PCR for verification of the *tlpB-hp0102* operon structure

For RT-PCR analysis, a DNaseI-digested RNA sample of *H. pylori* wild-type strain 26695 was reverse transcribed using the High Capacity cDNA Reverse Transcription Kit (Applied Biosystems by Thermo Fisher Scientific, #4368814) according to the manufacturer’s instruction. Briefly, reverse transcriptions were run with 1 µg of RNA ± MultiScribe™ Reverse Transcriptase using the following thermal cycling conditions: [25 °C, 10 min; 37 °C, 120 min; 85 °C, 5 min]. PCR reactions were performed using gene-specific primer sets for *tlpB* mRNA (JVO-5267/-5268), *hp0102* mRNA (CSO-0867/-0868), and for the *tlpB-hp0102* operon spanning junction (CSO-SP0061/-0062). PCR amplifications on gDNA of *H. pylori* wild-type strain 26695 served as a positive control. Subsequently, PCR products were run on a 2% agarose gel.

### Mouse infection studies

*H. pylori* strain X47-2AL wild-type strain and mutants were used to infect 5-weeks old NMRI-specific pathogen-free female mice (Charles River Laboratories) as published previously in ref. ^80^ and as described below. The housing conditions of the mice were optimal to reduce their stress. This included half/half light/dark cycle, a constant temperature of 20–23 °C, about 50% humidity, and *ad libitum* access to water and food. Before infection, the animals underwent an acclimation period of one week. For infections, about 10^8^ colony-forming units (CFUs) of *H. pylori* strains prepared in 100 μl of peptone broth were orogastrically administered to groups of seven mice per strain. In each experiment, four to five mice were inoculated with peptone broth alone as a naive mice negative control. One month after inoculation, mice were sacrificed and stomachs were crushed in peptone broth. CFUs of viable *H. pylori* bacteria per gram of stomach weight were calculated by serial dilutions and plating on blood-agar plates supplemented with the usual antibiotic-fungicide mixture with, in addition, 200 μg/ml bacitracin and 10 μg/ml nalidixic acid.

### SDS-PAGE and immunoblotting

Cells corresponding to an OD_600_ of 1 from *H. pylori* cells grown to mid-exponential growth phase were collected by centrifugation (16,000 × *g* at 4 °C for 2 min), resuspended in 100 µl 1× protein loading buffer (62.5 mM Tris-HCl pH 6.8, 100 mM DTT, 10% glycerol, 2% SDS, 0.01% bromophenol blue) and boiled at 95 °C for 8 min.

For SDS-PAGE, protein samples corresponding to a total OD_600_ of 0.1 were separated by 12% SDS-PAA gels and stained by Coomassie (Fermentas, #R0571). For western blot analysis, protein samples corresponding to an OD_600_ of 0.1 or 0.01 were separated by 10–12% SDS-PAGE and transferred to a PVDF membrane by semi-dry blotting. Membranes were blocked for 1 h with 10% milk powder/TBS-T and incubated overnight with primary antibody at 4 °C. After washing with TBS-T, membranes were incubated for 1 h with secondary antibody linked to horseradish peroxidase. Signals were detected using ECL-reagent and Image Quant LAS 4000.

To detect the four chemotaxis receptors TlpA, B, C, and D, a polyclonal rabbit TlpA22-antiserum (1:2,000 in 3%BSA/TBS-T) that recognizes a conserved cytosolic domain (kindly provided by Karen Ottemann, University of California, Santa Cruz, CA) and secondary goat anti-rabbit IgG (1:10,000 in 3% BSA/TBS-T; GE-Healthcare, #RPN4301, RRID: AB_2650489) were used. TlpB::3×FLAG was detected by a monoclonal anti-FLAG antibody (1:1,000 in 3% BSA/TBS-T; Sigma-Aldrich, #F1804, RRID: AB_262044) and secondary sheep anti-mouse IgG (1:10,000 in 3% BSA/TBS-T; GE-Healthcare, #RPN4201). GroEL, was visualized by monoclonal anti-GroEL antibody (1:10,000 in 3% BSA/TBS-T; Sigma-Aldrich, #G6532, RRID: AB_259939) and goat anti-rabbit IgG (1:10,000 in 3%BSA/TBS-T). GFP reporter fusion proteins were detected by an anti-GFP antibody (1:1,000 in 3% BSA/TBS-T; Roche, #11814460001) and secondary sheep anti-mouse IgG (1:10,000 in 3% BSA/TBS-T).

### LPS silver-staining and Lewis x antigen analysis

For LPS silver-staining, protein samples were digested with Proteinase K solution (20 mg/ml) for 90–180 min at 60 °C, followed by boiling of the samples for 10 min at 98 °C. Lipopolysaccharide structures from *H. pylori* were visualized by silver-staining according to ref. ^74^. Briefly, LPS samples corresponding to an OD_600_ of 0.1 were separated on 15% one dimensional SDS-PAA gel and fixed overnight in 25% isopropanol and 7% acetic acid (1× fixing solution). After incubation in sodium periodate solution (0.7% (w/v) Na_5_IO_6_ in 1× fixing solution) for 15 min, gels were washed with water (3 × 30 min). Silver staining solution (0.35% ammonia, 0.02 N NaOH, 0.4% (w/v) silver nitrate) was applied with vigorous agitation for 10 min. Following additional washing steps, gels were developed using a 2.5% (w/v) sodium carbonate solution containing 0.01% (v/v) formaldehyde (37%). Upon completion, 50 mM EDTA was used to stop the development.

For western blot analysis of Lewis x/y antigens, LPS samples corresponding to an OD_600_ of 0.02 were separated on 15% SDS-PAGE and transferred to a PVDF membrane by semi-dry blotting. Western blot analysis was performed using monoclonal anti-Lewis x antibody (1:500 in 3% BSA/TBS-T, Calbiochem, #434631) and monoclonal anti-Lewis y antibody (1:500 in 3% BSA/TBS-T, Merck, #434636). Chemiluminescence detection was performed with a secondary sheep anti-mouse IgG antibody conjugated to horseradish peroxidase (1:1,000 in 3% BSA/TBS-T, GE Healthcare, #RPN4201).

### Growth/survival under high salt conditions

*H. pylori* strains were grown in liquid medium (BHI) to exponential growth phase (OD_600_ of ~0.7–1.0). Cells were adjusted to an OD_600_ of 1.0 in 0.5 ml BHI and serial dilutions of the indicated strains were spotted on GC-agar plates containing sodium chloride at 85 mM (no stress, normal salt content), 200 mM (mild stress), or 260 mM (harsh stress). Plates were incubated for 3–5 days at 37 °C under microaerobic conditions.

### Antibiotics sensitivity tests

The MICs of *H. pylori* wild-type and mutant strains to polymyxin B were determined in triplicates by Epsilometer (E)-tests (bioMérieux, Inc., #533400) according to the manufacturer’s instructions. Briefly, *H. pylori* cells corresponding to an OD_600_ of 0.1 (exponential growth phase) were spread on GC-agar plates and E-stripes (polymyxin B concentration from 0.064 to 1024 µg/ml) were applied onto the dried surface of the agar plates. MIC values were read from the scale at the intersection of the elliptical zone of inhibition with the graded E-strip after 3 days of incubation under microaerobic conditions at 37 °C. E-test MICs that were between the standardized two-fold dilution steps were rounded to the next higher concentration.

### Biocomputational search for *repG*, *tlpB*, and *hp0102* homologs

Using BLAST (Basic Local Alignment Search Tool, NCBI) and SyntTax webserver (https://archaea.i2bc.paris-saclay.fr/SyntTax/), biocomputational searches for *repG*, *tlpB*, and *hp0102* homologs were performed in diverse *Helicobacter* species (spp.) and other related Epsilonproteobacteria, including *Campylobacter* spp., *Wolinella* spp., *Sulfuricurvum* spp., *Arcobacter* spp., *Sulfurospirillum* spp., and *Nautilia profundicola* (~120 genomes). Genes were considered to be homologous when they share more than 30% percent sequence similarity with *repG*, *tlpB*, and *hp0102* of *H. pylori* strain 26695. No homologs for *repG, tlpB*, and *hp0102* could be identified outside of the *Helicobacter* spp. and thus, are not further mentioned in the main text.

### Ethics Statement

Experiments in mice were carried out in strict accordance with the recommendations in the Specific Guide for the Care and the Use of Laboratory Animals of the Institut Pasteur, according to the European Directive (2010/63/UE) and the corresponding French law on animal experimentation (Arrêtés de 1988). The protocol has been approved by the Committee of Central Animal Facility Board of the Institut Pasteur. To follow the new European directives, the project was approved by the CETEA, Comité d’éthique en Expérimentation Animale of the Institut Pasteur (#2013-0051) and by the Ministère de l’Enseignement Supérieur et de la recherche (#751501).

### Reporting summary

Further information on research design is available in the Nature Research Reporting Summary linked to this article.

## Supplementary information

Supplementary Information

Reporting Summary

## Data Availability

All data supporting the findings of this study are available within the article and its Supplementary information files. All data is available from the corresponding author upon reasonable request. Source data are provided with this paper.

## Source data

Source Data
