# Supplementary material for: Small RNA mediated gradual control of lipopolysaccharide biosynthesis affects antibiotic resistance in Helicobacter pylori
Source: Nat Commun. 2021 Jul 21;12:4433. doi: 10.1038/s41467-021-24689-2 (PMC8295292; doi:10.1038/s41467-021-24689-2)
Supplement: Supplementary file 1 — Supplementary Information [file 41467_2021_24689_MOESM1_ESM.pdf]

Supplementary Material

Small RNA mediated gradual control of lipopolysaccharide biosynthesis affects antibiotics resistance in *Helicobacter pylori*

Sandy R. Pernitzsch, Mona Alzheimer, Belinda U. Bremer, Marie Robbe-Saule, Hilde de Reuse, and Cynthia M. Sharma

This supplement contains:

Supplementary Figures 1 to 9
Supplementary Tables 1 to 8
Supplementary References

Supplementary Figures

Supplementary Figure 1

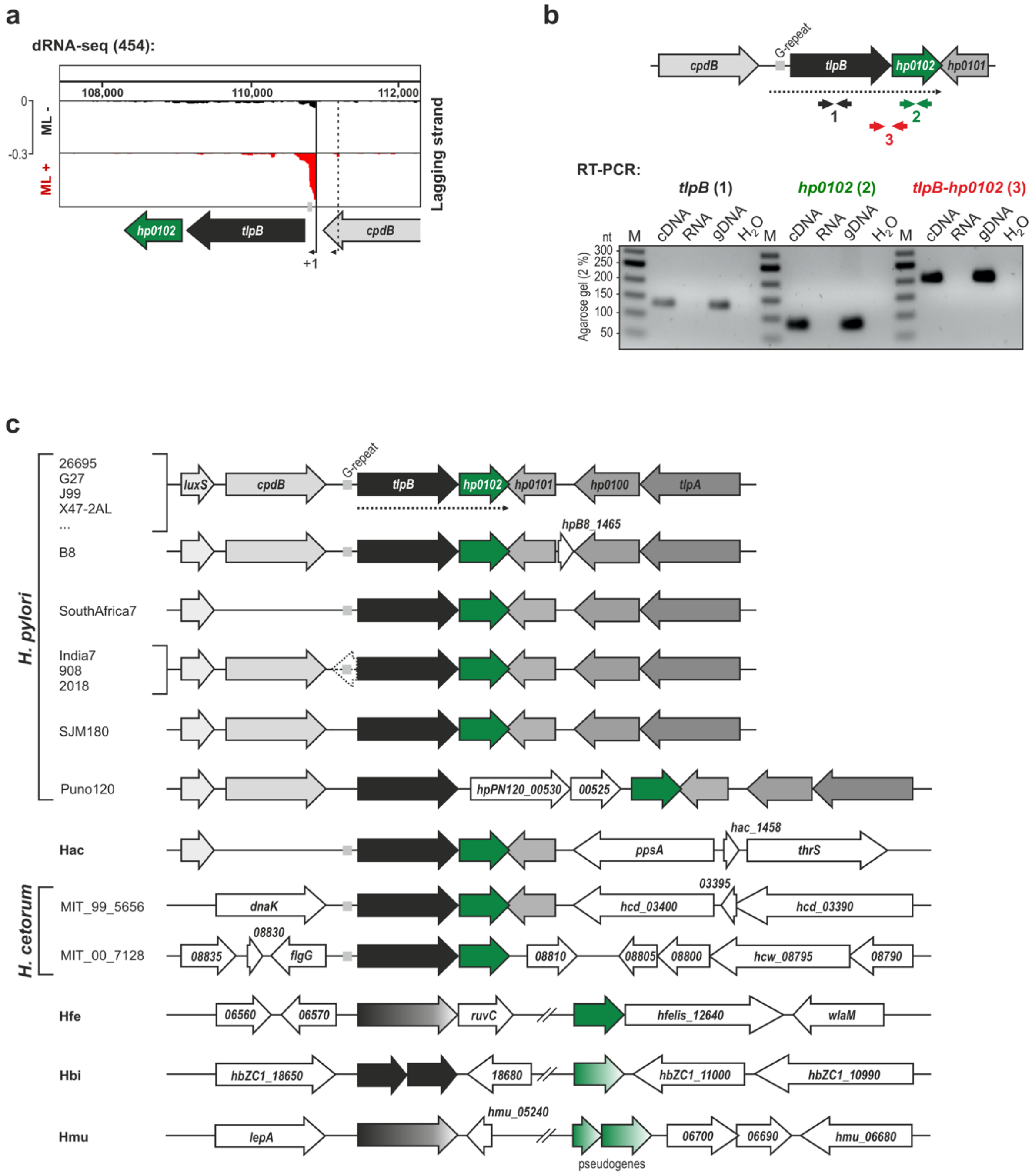

Supplementary Figure 1: Conservation of the *tlpB-hp0102* operon among *Helicobacter* strains and species. (a) Screenshot of a genome browser showing cDNA read coverage at the bicistronic *tlpB-hp0102* operon based on our previous differential RNA-seq (dRNA-seq) analysis of *H. pylori* 26695[1]. RNA samples isolated from cells grown to mid-exponential growth phase (ML) were used for

preparation of two cDNA libraries: (-) RNA left untreated, (+) RNA treated with terminator nuclease to enrich for primary transcripts. Thick arrows represent annotated open reading frames (ORFs) and thin arrows transcriptional start sites (TSS; +1; filled line - primary TSS, dotted line - secondary TSS). **(b)** *(Upper panel)* Schematic representation of the bicistronic *tlpB-hp0102* operon and RT-PCR validation strategy. Small arrows represent locations of gene-specific primers for *tlpB* (black), *hp0102* (green), and overlapping both genes *tlpB-hp0102* (red). RT-PCR assays were performed on total RNA isolated from strain 26695. *(Lower panel)* Agarose gel of RT-PCR reactions performed in the presence (cDNA) and absence (RNA) of reverse transcriptase. Control reactions using genomic DNA (gDNA) of 26695 and water (H_2O) are indicated. Lane M contains a 50 bp DNA ladder. Underlying source data are provided as a Source Data file. **(c)** Gene synteny analysis of the *tlpB-hp0102* operon (dotted line) in different gastric *Helicobacter* strains and species. Homologs are indicated by the same colors (Hac - *H. acinonychis,* Hce - *H. cetorum,* Hfe - *H. felis*, Hbi - *H. bizzozeronii*, Hmu - *H. mustelae*), unrelated genes are indicated in white. The homopolymeric G-repeat in the 5' UTR of the *tlpB* mRNA is represented by a gray box. A potential ORF (dotted, white arrow) is annotated in the *tlpB* 5' UTR in *H. pylori* strains India7, 908, and 2018. Gradually colored arrows indicate limited sequence homology, whereas fully colored arrows represent homologs with >50 % sequence conservation. In *H. pylori* strain Puno120, two genes belonging to a type-II DNA restriction and modification system are inserted downstream of *tlpB.*

Supplementary Figure 2

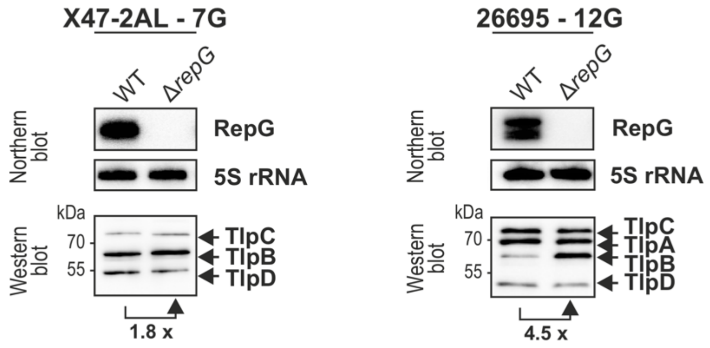

Supplementary Figure 2: Analysis of RepG-mediated TlpB regulation in different *H. pylori* strains. Total RNA and protein samples of X47-2AL and 26695 WT and Δ*repG* were harvested in exponential growth phase and were analyzed by northern and western blot, respectively. RepG was detected by radioactively labeled DNA oligonucleotide CSO-0003; 5S rRNA served as loading control. The chemotaxis receptors TlpA, TlpB, TlpC, and TlpD were detected by polyclonal anti-TlpA22 serum. The results shown are representative of at least three independent experiments. Underlying source data are provided as a Source Data file.

Supplementary Figure 3

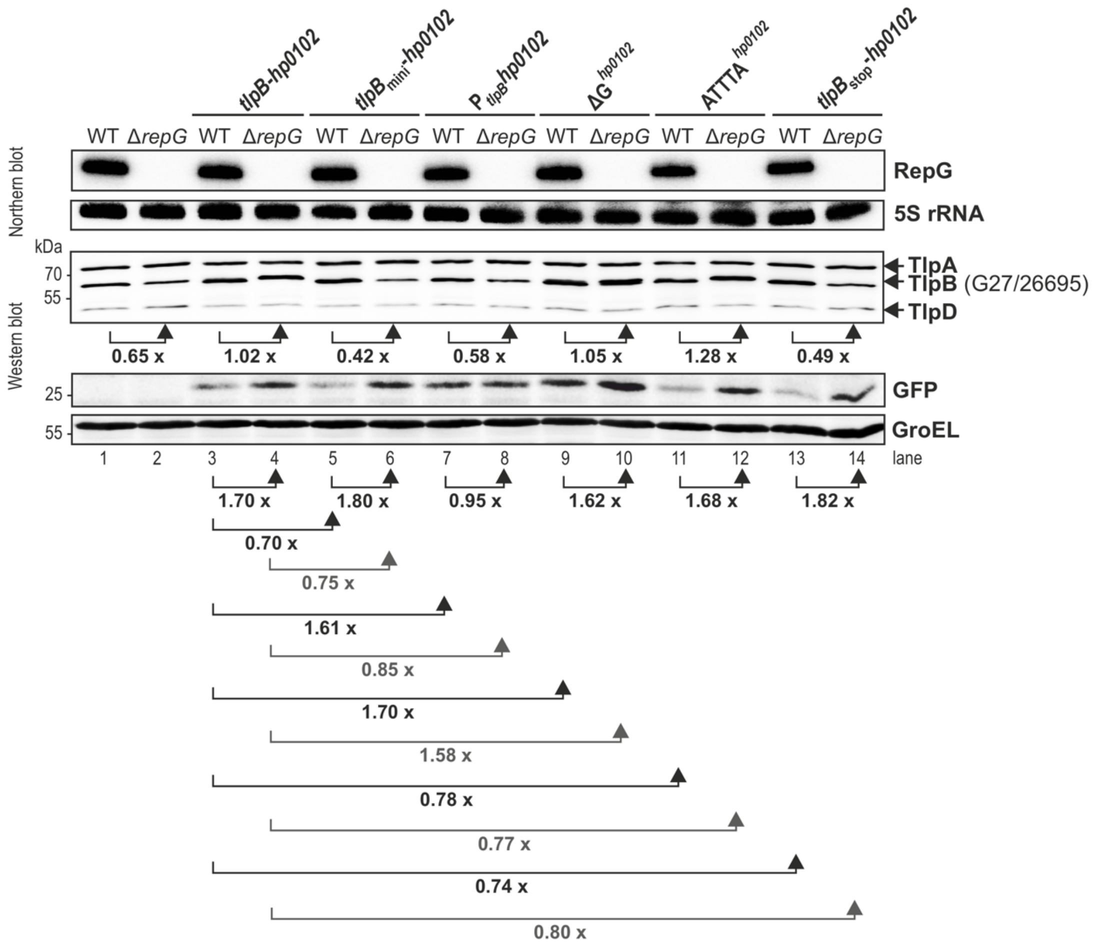

Supplementary Figure 3: Base-pairing of RepG to the homopolymeric G-repeat in the 5' UTR of the *tlpB-hp0102* mRNA results in repression of *hp0102* at the protein level. Several *hp0102*::*gfpmut3* reporter fusions (see also main Figure 2a; derived from *H. pylori* 26695) were investigated in *H. pylori* strain G27. *H. pylori* G27 wildtype, Δ*repG*, as well as WT and Δ*repG* strains carrying indicated *hp0102*::*gfpmut3* reporter fusions were grown to exponential phase, and RNA and protein samples were analyzed by northern (RepG, CSO-0003) and western blot (HP0102::GFP with anti-GFP antibody and TlpB with anti-TlpA22 antiserum), respectively. GroEL served as a loading control (anti-GroEL antibody). Depending on the G-repeat length, RepG mediates both activation of the endogenous G27 *tlpB* (14G) and repression of the ectopic *tlpB* of the 26695 (12G) reporter construct[2]. Accordingly, TlpB levels in the strains carrying the *tlpB*-*hp0102* (lanes 3-4), ΔG^{hp0102} (lanes 9-10) or $ATTTA^{hp0102}$ (lanes 11-12) fusions are the sum of both the endogenous G27 TlpB and ectopically expressed 26695 TlpB levels. The results shown are representative of at least three independent experiments. The joint result of all three replicates is shown in the bar diagram in Figure 2b. Source data underlying this figure is provided as a Source Data file.

Supplementary Figure 4

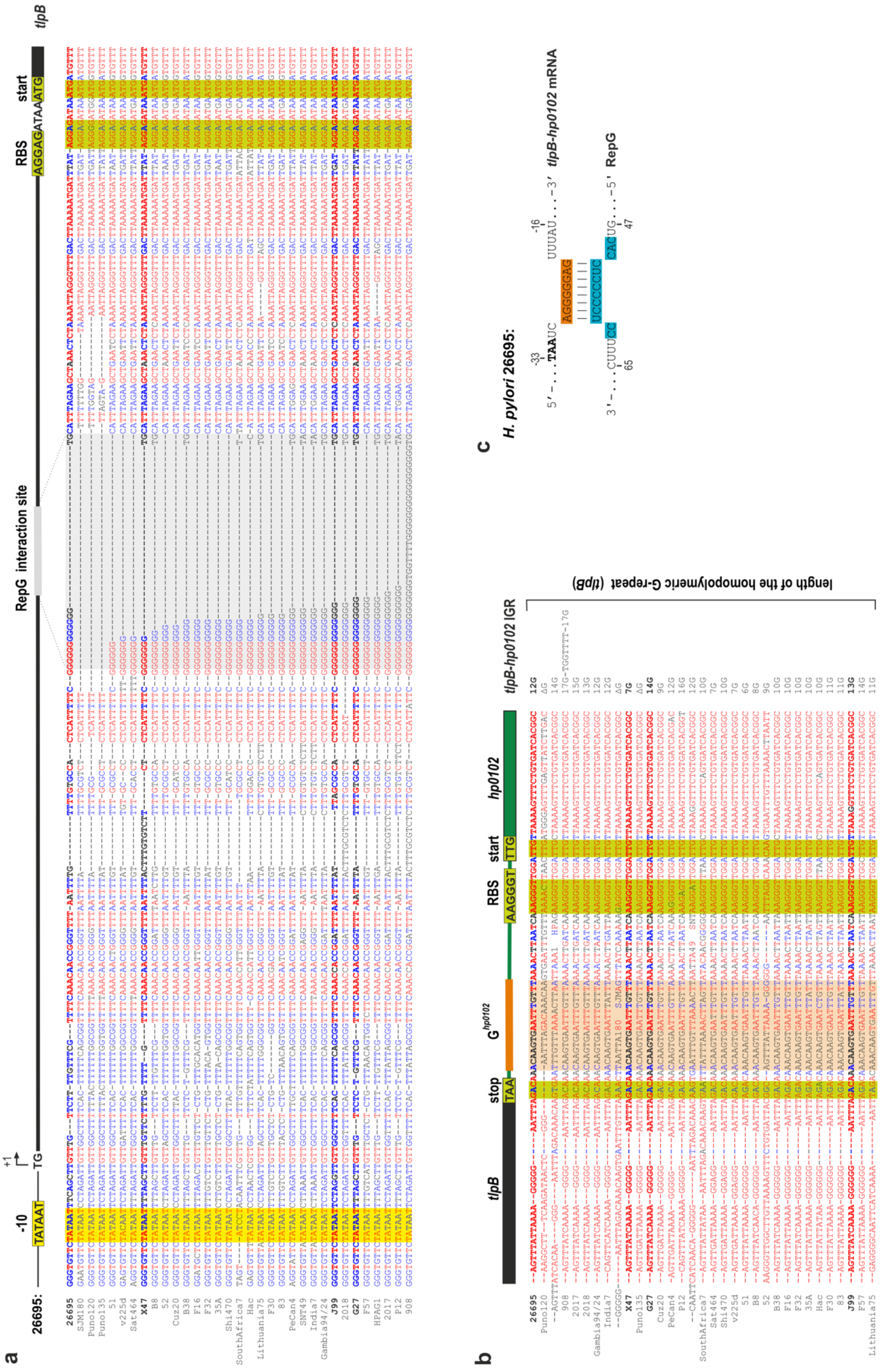

Supplementary Figure 4: Sequence alignment of the promoter, 5' UTR, and first amino acids of the *tlpB* coding region as well as the *tlpB-hp0102* intergenic region (IGR) in diverse *H. pylori* strains and *H. acinonychis* (Hac). (a-b) The *tlpB* stop codon, putative ribosome binding site (RBS), and annotated start codons of *tlpB* and *hp0102* are highlighted in light green. Potential RepG sRNA interaction sites (homopolymeric G-repeat and G^{hp0102}) are boxed in gray and orange, respectively. RepG-mediated *tlpB-hp0102* co-regulation was investigated in the strains marked in bold. **(c)** In *H. pylori* 26695, RepG was predicted to potentially base-pair to G^{hp0102} (orange) in the *tlpB-hp0102* IGR. The *tlpB* stop codon is marked in bold. Numbers indicate positions relative to the *hp0102* start codon. The previously identified *tlpB* binding site in the RepG terminator loop is shown in blue.

Supplementary Figure 5

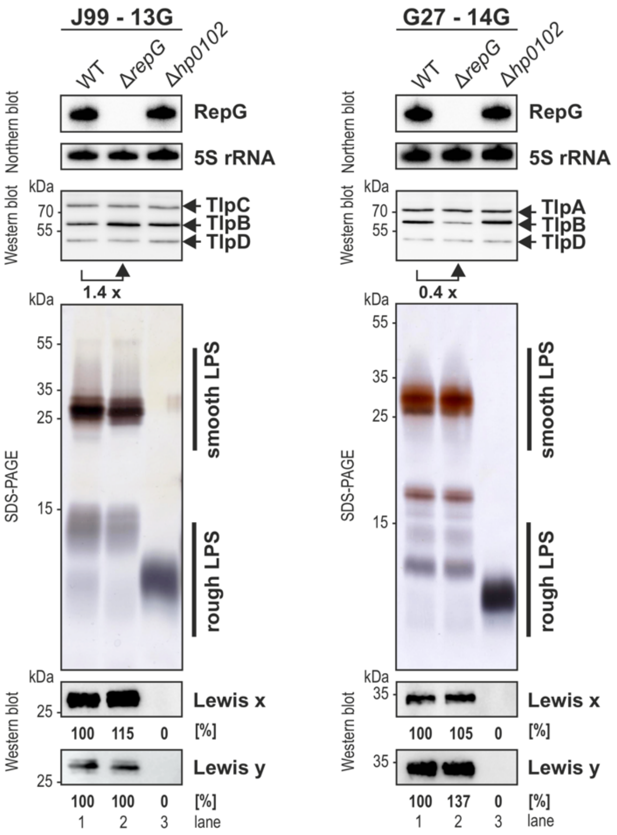

Supplementary Figure 5: HP0102 is involved in smooth LPS production in various *H. pylori* strains. Wildtype, Δ*repG*, and Δ*hp0102* deletion mutants of *H. pylori* strains J99 and G27 were grown to exponential growth phase. Total RNA and protein samples were analyzed by northern and western blot, respectively. RepG and the chemotaxis receptors TlpA, TlpB, TlpC, and TlpD were detected using a polyclonal anti-TlpA22 antiserum. *H. pylori* strains J99 and G27 each express only three chemotaxis receptors. LPS patterns and Lewis x/y antigen expression were analyzed by silver staining and western blot using a Lewis x/y antigen-specific antibody, respectively. The results shown are representative of at least three independent experiments. Source data underlying this figure is provided as a Source Data file.

Supplementary Figure 6

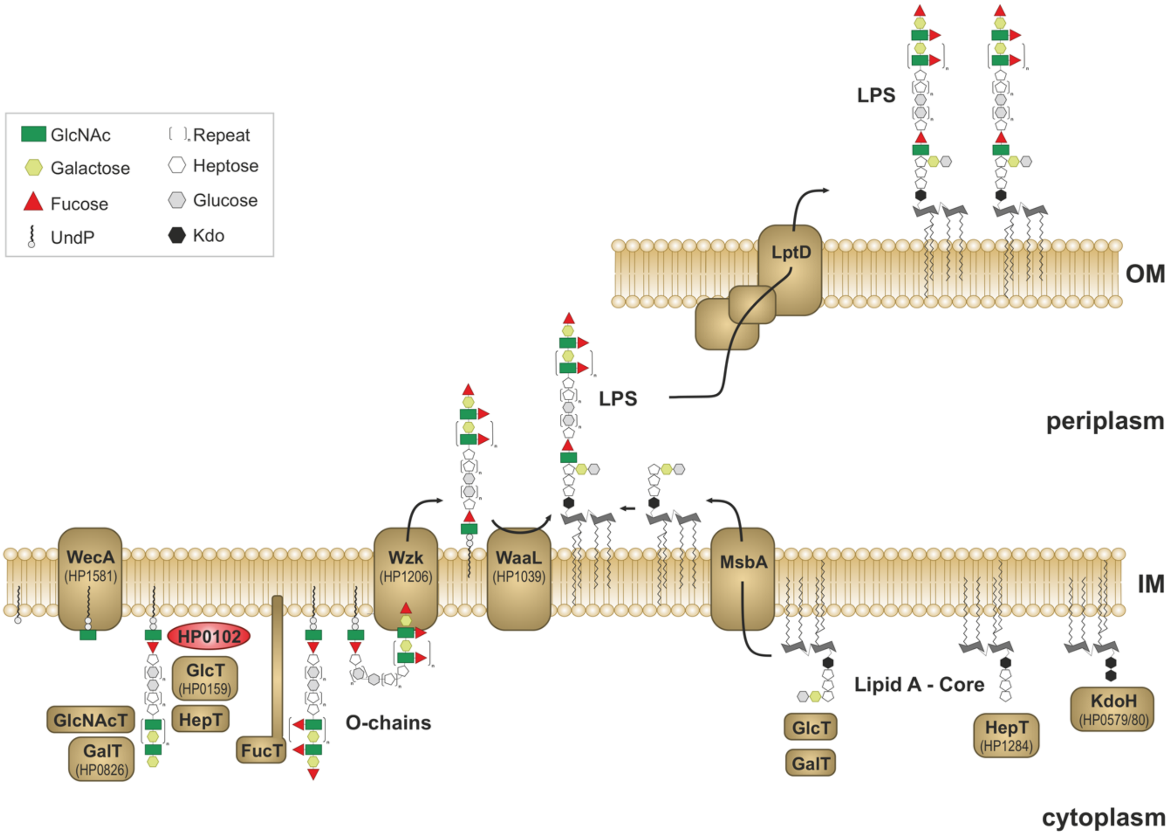

Schematic of the LPS biosynthesis pathway in *H. pylori*. Summary of the LPS biosynthesis pathway in *H. pylori* based on literature[3,4] and results from this study. The lipid A-core (right) and O-chains (left) are assembled independently from each other in the cytoplasm. Lipid A-core: Heptose and glucose units are added to the lipid A by multiple heptosyltransferases (HepTs) and glucosyltransferases (GlcTs). O-chains: The O-chains are assembled onto a polyisoprenoid membrane anchor (UndP). The initiating glycosyltransferase WecA transfers an N-acetylglucosamine unit from a nucleotide-activated donor (uridine diphosphate (UDP)-GlcNAc) to the lipid carrier, providing the platform for the O-chain synthesis. The linear O-chain backbone is assembled by processive galactosyltransferases (GalTs) and N-acetylglucosaminyltransferases (GlcNAcTs). Fucosyltransferases (FucTs) attach fucose units to the O-chain backbone, generating Lewis antigens. O-chains and the lipid A-core moiety are translocated through the inner membrane (IM) by the flippase Wzk and transmembrane protein MsbA, respectively, and are fused together by the O-chain ligase WaaL. The LPS molecule is then transported to the outer leaflet of the outer membrane (OM). Our results together with recent observations by Li et al. [4] indicate that HP0102 is the fucosyltransferase involved in the biosynthesis of the conserved trisaccharide of the O-antigen. See also Figure S7.

Supplementary Figure 7

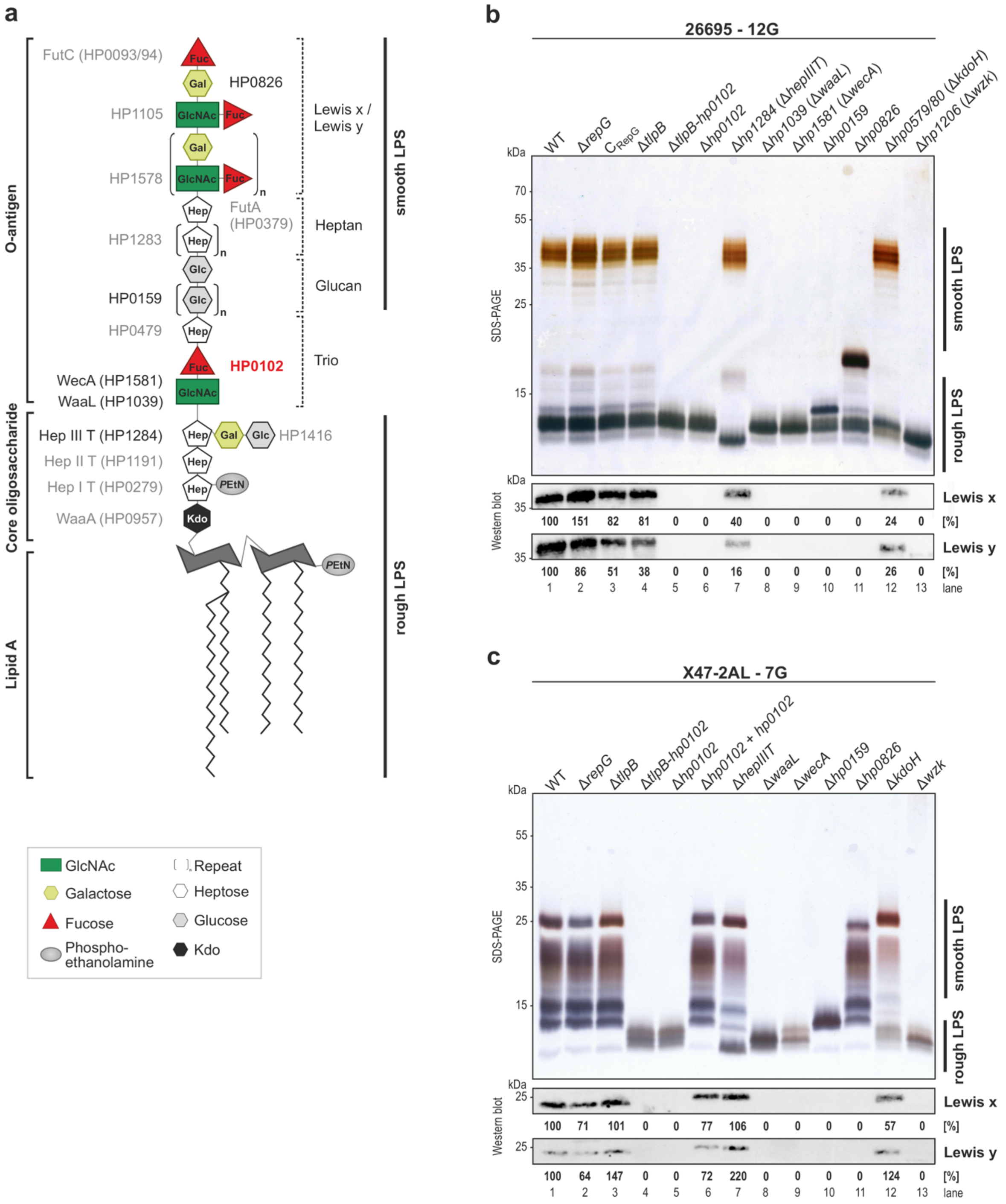

Supplementary Figure 7: Comparison of LPS profiles elicited by deletion *hp0102* and additional genes involved in LPS biosynthesis in *H. pylori* strains 26695 and X47-2AL. (a) Schematic representation of the *H. pylori* 26695 LPS structure and genes involved in its synthesis according to and illustration inspired by Li et al. (2019)[4]. LPS samples harvested from WT and indicated mutant strains of *H. pylori* strains 26695 **(b)** and X47-2AL **(c)** during exponential growth were separated on 15 % SDS-PAGE gels and either directly visualized by silver staining, or electro-blotted to PVDF membranes and

probed with Lewis x/y antigen-specific antibodies. The results shown are representative of at least three independent experiments. Source data underlying b and c are provided as a Source Data file.

Supplementary Figure 8

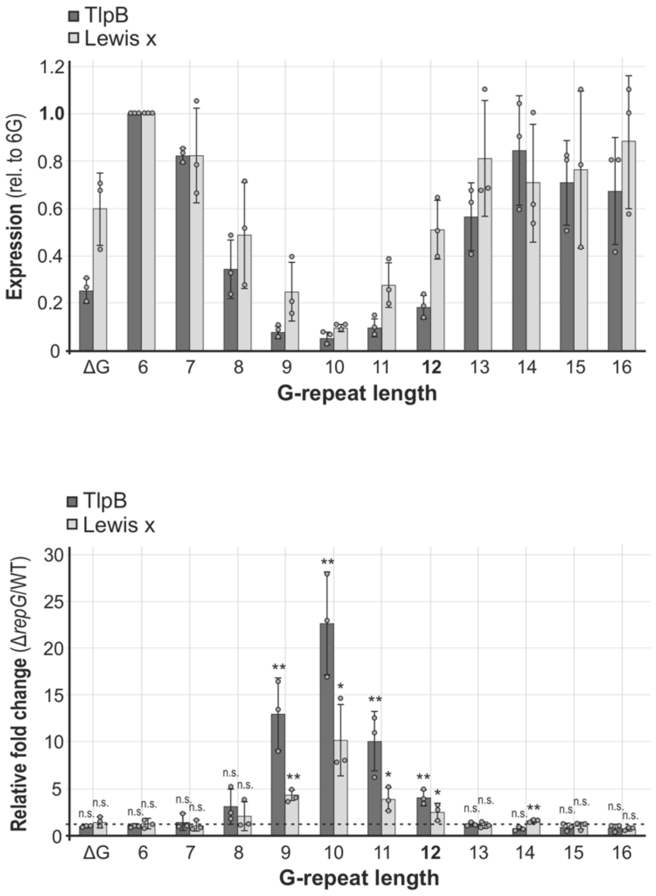

Supplementary Figure 8: The length of the homopolymeric G-repeat in the *tlpB* mRNA leader impacts RepG-mediated TlpB and HP0102 regulation, and in turn Lewis x antigen expression. (*Upper panel*) Quantification of relative TlpB protein and Lewis x antigen levels in the different *tlpB* leader mutants of *H. pylori* strain 26695 in the wild-type background grown to exponential growth phase. TlpB protein and Lewis x antigen levels in the *tlpB* 6G-leader variant were used as reference and set to 1. (*Lower panel*) Relative fold change of TlpB protein and Lewis x antigen levels upon *repG* deletion in *tlpB* leader mutants when compared to the respective wild-type backgrounds. Bars represent the mean determinations of triplicate measurements (n = 3) ± standard deviation (s.d.) derived from western blot quantification (see main Figure 5b). ** - significant, p-value < 0.01; * - significant, p-value < 0.05; n.s., not significant; Student's t-test, two-tailed. Source data underlying this figure is provided as a Source Data file.

Supplementary Figure 9

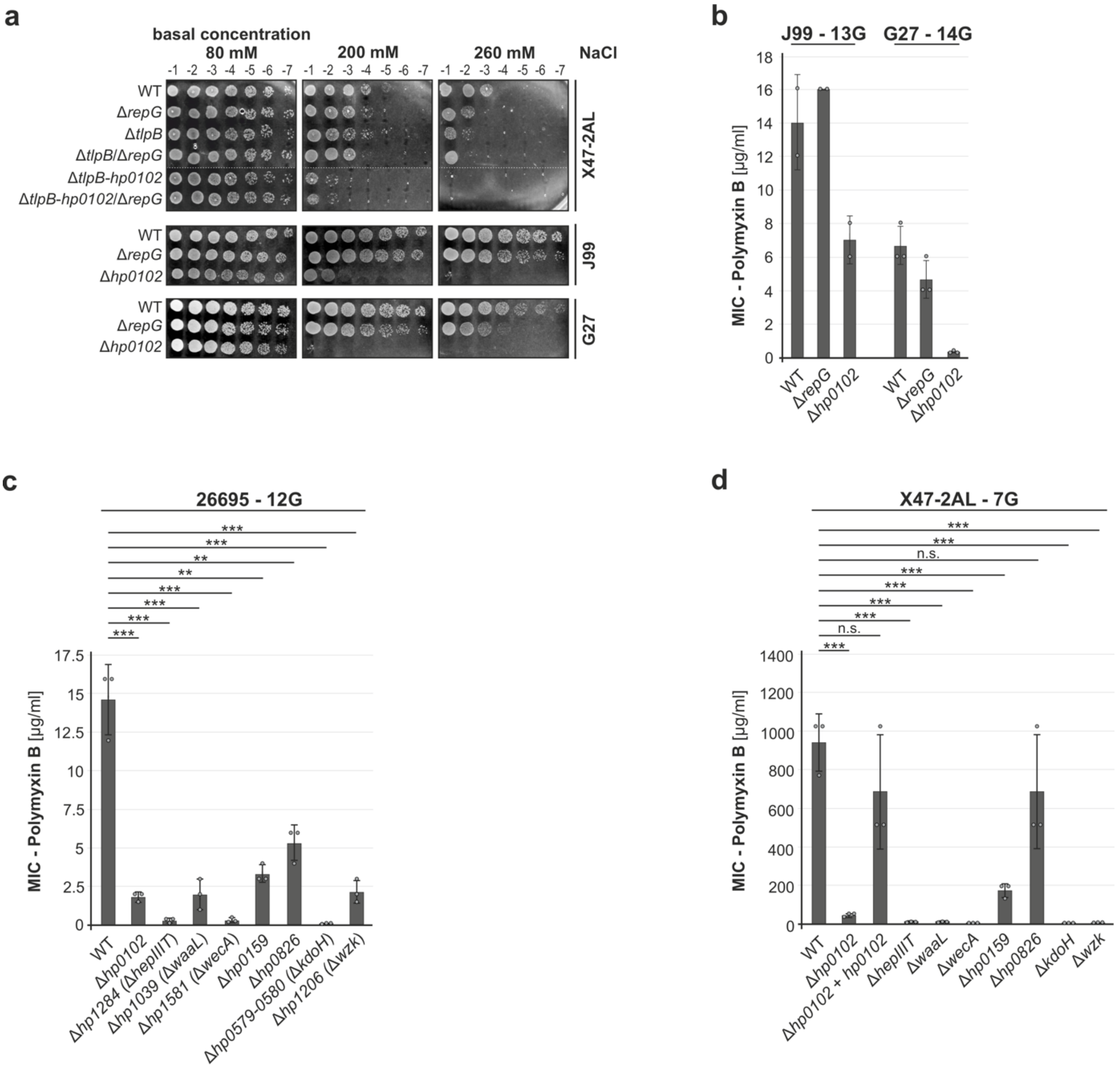

Supplementary Figure 9: *H. pylori* Δ*hp0102* mutant strains show increased susceptibility to high salt-induced membrane stress and to polymyxin B. (a) Growth of wild-type and indicated mutant strains of *H. pylori* X47-2AL, J99, and G27 on GC-agar plates without (80 mM, basal concentration) or with increased NaCl concentrations (200 mM, 260 mM). Ten-fold dilutions of the bacterial suspensions were spotted on indicated GC-agar plates and incubated for 3 to 5 days at 37 °C under microaerobic conditions. The results shown are representative of at least two biological replicates. **(b-d)** Antibiotic sensitivity testing using E-tests (bioMérieux, Inc.) for *H. pylori* strains J99 and G27 (b), X47-2AL (c), and 26695 (d). *H. pylori* cells corresponding to an OD_{600nm} of 0.01 were plated on GC-agar plates and E-test stripes (0.064-1024 µg/ml polymyxin B) were placed on top. MICs were determined after 3 days of incubation under microaerobic conditions. Bars represent the mean determinations of triplicate measurements of MICs (n = 3, exception: n = 2 for J99) ± standard deviations (s.d.). *** - highly significant, p-value < 0.001; ** - very significant, p-value < 0.01; n.s., not significant; Student's t-test, two-tailed. Source data underlying b, c, and d are provided as a Source Data file.

Supplemental Tables

Supplementary Table 1: Minimal inhibitory concentrations (MICs) of *H. pylori* 26695, X47-2AL, J99, and G27 wild-type and mutant strains against polymyxin B. MICs against polymyxin B (PxB) were determined using Epsilometer (E)-tests (bioMérieux) in three biological replicates and the averages are given in µg/ml with the corresponding standard deviations (s.d.). Fold changes of *H. pylori* mutant strains were calculated relative to their respective parental wildtype. Positive (+) fold changes indicate an increased resistance to polymyxin B compared to WT, negative (-) fold changes represent decreased resistance (-xx-fold more sensitive compared to WT; +xx-fold less sensitive compared to WT).

**Strains**	**Role in LPS biosynthesis**	**PxB MIC** [µg/ml]	**Fold change** (relative to WT)
***H. pylori* strain 26695**			
WT	full-length LPS	14.7 ± 2.3	
Δ*repG*		29.3 ± 4.6	+2.0
C_{RepG}		14.7 ± 2.3	–
Δ*tlpB*		21.3 ± 4.6	+1.5
Δ*tlpB-hp0102*		2.0 ± 0.0	-7.2
Δ*hp0102*	Trio fucosyltransferase	1.8 ± 0.3	-7.8
Δ*hp1284* (Hep III T)	Hep III transferase	0.3 ± 0.1	-48.7
Δ*hp1039* (WaaL)	O-antigen ligase	2.0 ± 1.0	-7.0
Δ*hp1581* (WecA)	O-antigen initiating ligase	0.3 ± 0.2	-45.9
Δ*hp0159*	Glycosyltransferase	3.3 ± 0.6	-4.3
Δ*hp0826*	Galactosyltransferase	5.3 ± 1.2	-2.7
Δ*hp0579-580*	KDO hydrolase	0.1 ± 0.035	-137.3
Δ*hp1206* (Wzk)	O-antigen flippase	2.2 ± 0.8	-6.6
***H. pylori* strain X47-2AL**			
WT	full-length LPS	938.7 ± 147.8	
Δ*repG*		682.7 ± 147.8	-1.4
Δ*tlpB*		768.0 ± 256.0	-1.2
Δ*tlpB-hp0102*		32.0 ± 0.0	-29.3
Δ*hp0102*	Trio fucosyltransferase	42.7 ± 9.2	-22.0
Δ*hp0102 + hp0102*		682.7 ± 295.6	-1.4
Δ*hp1284* (Hep III T)	Hep III transferase	6.0 ± 2.0	-156.4
Δ*hp1039* (WaaL)	O-antigen ligase	6.0 ± 2.0	-156.4
Δ*hp1581* (WecA)	O-antigen initiating ligase	0.4 ± 0.2	-2,631.8
Δ*hp0159*	Glycosyltransferase	170.7 ± 37.0	-5.5
Δ*hp0826*	Galactosyltransferase	682.7 ± 295.6	-1.4
Δ*hp0579-580*	KDO hydrolase	0.1 ± 0.043	-16,822.0
Δ*hp1206* (Wzk)	O-antigen flippase	2.5 ± 0.9	-375.5
***H. pylori* strain J99**			
WT	full-length LPS	14.0 ± 2.0	
Δ*repG*		14.0 ± 2.0	–
Δ*hp0102*	Trio fucosyltransferase	7.3 ± 1.5	-1.9
***H. pylori* strain G27**			
WT	full-length LPS	7.0 ± 1.4	
Δ*repG*		5.0 ± 1.4	-1.4
Δ*hp0102*	Trio fucosyltransferase	0.3 ± 0.09	-23.3

Supplementary Table 2: SSRs associated with genes involved in LPS biosynthesis in *H. pylori* strain 26695

***H. pylori* 26695 – homopolymeric repeats**					
**Promoter**		**5' UTR**		**Coding sequence**	
**ID (SSR)**	**Function**	**ID (SSR)**	**Function**	**ID (SSR)**	**Function**
hp0651 (A7)	*futB*, fucosyltransferase	*hp0208* (A11[b])	*rfaJ2*, glucosyltransferase	*hp0093-94* (C14)	*fucC*, fucosyltransferase
		***hp0102* (G12[a])**	**fucosyltransferase**	*hp0208* (AG11)	*rfaJ2*, glycosyltranferase
		hp1105 (A15)	N-acetyl-glycosamyltransferase	*hp0217* (G12)	N-acetylgalactosaminyltransferase
				hp0379 (C13)	*futA,* fucosyltransferase
				hp0580 (C8)	Kdo-hydrolase
				hp0619 (C13)	*lex2B*, glycosyltransferase
				hp0651 (C13)	*futB*, fucosyltransferase
				hp1417 (AG9)	hypothetical protein[c]

[a]*hp0102* is located in an operon with *tlpB*, G12 in the 5' UTR of the *tlpB* mRNA

[b]*hp0208* does not have a defined TSS, but RNA-seq indicated a potential 110-nt long 5' UTR (Pernitzsch and Sharma, unpublished)

[c]*hp1417* is potentially involved in LPS core assembly[3]

Supplementary Table 3: G-repeat length in the *tlpB* 5' UTR of sequential *H. pylori* isolates from human or after re-isolation from animals. Lines frame *H. pylori* isolates that were obtained from the same patient or that were re-isolated after mice/gerbil infection studies. The lengths of the G-repeats in the *tlpB* 5' UTRs of isolates from Narino, Colombia, from the study of Kennemann *et al.* (2011)[5] were re-sequenced by Sanger sequencing.

Strain	NCBI Acc. No	Time scale	G-repeat length	G-repeat variation	Origin/Comment	Reference
**908**	NC_017357		17G-TGGTTTT-17G		West African duodenal ulcer disease patient in France	Ref[6]
**2017**	NC_017374	10 yrs	15G	yes	Re-isolate from antrum	Ref[7]
**2018**	NC_017381	10 yrs	13G	yes	Re-isolate from corpus	
**NQ367**	NZ_CADL00000000		15/16G°		Isolates from Narino, Colombia	Ref[5]
**NQ1671**	NZ_CADM00000000	3 yrs	13G	yes		
**NQ4191**	NZ_CADN00000000	16 yrs	13G	yes		
**NQ392**	NZ_CADI00000000		13G°		Isolates from Narino, Colombia	Ref[5]
**NQ1707**	NZ_CADJ00000000	3 yrs	14G	yes		
**NQ4060**	NZ_CADK00000000	16 yrs	15/16G°	yes		
**NQ315**	NZ_CADE00000000		12G		Isolates from Narino, Colombia	Ref[5]
**NQ1712**	NZ_CADF00000000	3 yrs	13G	yes		
**NQ352**	NZ_CADG00000000		12G°		Isolates from Narino, Colombia	Ref[5]
**NQ1701**	NZ_CADH00000000	3 yrs	14G°	no		
**BM012A**	NZ_CP006888.1		12G		Isolate from asymptomatic human	Ref[8]
**BM012B**	NZ_CP007605.1	44 d	11G	yes	Isolate after re-infection of cured patient with strain BM012A	
**BM012S**	NZ_CP006889.1	5 mo	10G	yes	Spouse of patient with BM012A, natural transmission between spouses (timepoint of transmission unclear)	
**BM013A**	NZ_CP007604.1		12G		Isolate from asymptomatic human	Ref[8]
**BM013B**	NZ_CP007606.1	20 d	12G	no	Isolate after re-infection of cured patient with strain BM013A	

**BCM-300**	NZ_LT837687.1		14G		*cagA*-positive *H. pylori* strain	Ref[9]
**HE136/09**	NZ_LT635473.1	12 wk	14G	no	Re-isolates from five patients infected with BCM-300, placebo group	
**HE141/09**	NZ_LT635471.1	12 wk	15G	yes		
**HE143/09**	NZ_LT635458.1	12 wk	14G	no		
**HE147/09**	NZ_LT635477.1	12 wk	14G	no		
**HE170/09**	NZ_LT635472.1	12 wk	14G	no		
**HE93/10**	NZ_LT838273.1	12 wk	13G	yes	Re-isolates from seven patients infected with BCM-300, patients were previously challenged with prophylactic vaccine candidate	
**HE101/09**	NZ_LT635456.1	12 wk	14G	no		
**HE132/09**	NZ_LT635459.1	12 wk	15G	yes		
**HE134/09**	NZ_LT635476.1	12 wk	14G	no		
**HE142/09**	NZ_LT635478.1	12 wk	14G	no		
**HE171/09**	NZ_LT635474.1	12 wk	14G	no		
**HE178/09**	NZ_LT635460.1	12 wk	13G	yes		
**Hp141**			12G-TGC#		Woman with gastritis in Poitiers, France	Ref[10]
**HP141***		150 d	10G-C#	yes	Re-isolate from femal C57BL/6 inbred mice	
**HP145**			10G		Woman with prepyloric ulcer in Poitiers, France	Ref[10]
**HP145***		150 d	10G	no	Re-isolate from femal C57BL/6 inbred mice	
**HP87**			13G		Original human isolate	Ref[11]
**HP87 P7***			16/17/18G	yes	Gerbil adapted strain	
**HP87 P7 *tlpD***			16/17/18G		*tlpD* mutant of gerbil adapted strain	Ref[11]
**HP87 P7 *tlpD* RI**		6 wk	16/17/18G		Re-isolate from gerbil antrum	

° The G-repeat length determined by Sanger sequencing differed from the genome sequence determined by 454- sequencing.

* *H. pylori* isolates that were re-isolated from C57BL/6 inbred mice or gerbils.

Additional nucleotide variations that were identified in the flanking region of the homopolymeric G-repeat.

Supplementary Table 4: Bacterial strains

Name	Description	Strain number	*H. pylori*	Resistance
**WT / 26695**	Wildtype (NCBI Acc-no. NC_000915), kindly provided by T. F. Meyer (MPI-IB, Berlin, Germany); Ref[12]	CSS-0004	26695	
[a]**Δ*repG***	*repG*::*aphA*-3	JVS-7014	26695	Kan^{R}
[a]**C$_{RepG}$**	*repG*::*aphA*-3, *rdxA*::*repG*:*catGC*	CSS-0046	26695	Kan^{R} Cm^{R}
[a]**SL 2**	*repG*::*aphA*-3, *rdxA*::*repG*-SL 2:*catGC*	CSS-0747	26695	Kan^{R} Cm^{R}
[a]**ΔCU**	*repG*::*aphA*-3, *rdxA*::*repG*-ΔCU:*catGC*	CSS-0157	26695	Kan^{R} Cm^{R}
[a]**Δ*tlpB***	*tlpB*::*rpsL-erm*	CSS-0163	26695	Erm^{R}
**Δ*hp0102***	*hp0102*::*rpsL-erm*	CSS-1000	26695	Erm^{R}
**Δ*tlpB* (Kan)**	*tlpB*::*aphA*-3	CSS-5924	26695	Kan^{R}
**Δ*hp0102* (Kan)**	*hp0102*::*aphA*-3	CSS-5942	26695	Kan^{R}
**Δ*tlpB-hp0102***	*tlpB-hp0102*::*aphA*-3	CSS-5926	26695	Kan^{R}
**Δ*hp1284***	*hp1284*::*aphA*-3	CSS-5928	26695	Kan^{R}
**Δ*hp1039***	*hp1039*::*aphA*-3	CSS-5930	26695	Kan^{R}
**Δ*hp1581***	*hp1581*::*aphA*-3	CSS-5932	26695	Kan^{R}
**Δ*hp0159***	*hp0159*::*aphA*-3	CSS-5934	26695	Kan^{R}
**Δ*hp0826***	*hp0826*::*aphA*-3	CSS-5936	26695	Kan^{R}
**Δ*hp0579-0580***	*hp0579-0580*::*aphA*-3	CSS-5938	26695	Kan^{R}
**Δ*hp1206***	*hp1206*::*aphA*-3	CSS-5940	26695	Kan^{R}
[a]***tlpB*::3xFLAG*/ *tlpB* ΔG**	*rpsL-str^{R}*, *tlpB*::*tlpB*-3xFLAG*, *tlpB* ΔG::*tlpB*:*rpsL-erm*	CSS-0471	26695 Str^{R}	Str^{R} Erm^{R}
[a]***tlpB*::3xFLAG*/ *tlpB* 6G**	*rpsL-str^{R}*, *tlpB*::*tlpB*-3xFLAG*, *tlpB* 6G::*tlpB*:*rpsL-erm*	CSS-0472	26695 Str^{R}	Str^{R} Erm^{R}
[a]***tlpB*::3xFLAG*/ *tlpB* 7G**	*rpsL-str^{R}*, *tlpB*::*tlpB*-3xFLAG*, *tlpB* 7G::*tlpB*:*rpsL-erm*	CSS-0473	26695 Str^{R}	Str^{R} Erm^{R}
[a]***tlpB*::3xFLAG*/ *tlpB* 8G**	*rpsL-str^{R}*, *tlpB*::*tlpB*-3xFLAG*, *tlpB* 8G::*tlpB*:*rpsL-erm*	CSS-0474	26695 Str^{R}	Str^{R} Erm^{R}
[a]***tlpB*::3xFLAG*/ *tlpB* 9G**	*rpsL-str^{R}*, *tlpB*::*tlpB*-3xFLAG*, *tlpB* 9G::*tlpB*:*rpsL-erm*	CSS-0475	26695 Str^{R}	Str^{R} Erm^{R}
[a]***tlpB*-3xFLAG*/ *tlpB* 10G**	*rpsL-str^{R}*, *tlpB*::*tlpB*-3xFLAG*, *tlpB* 10G::*tlpB*:*rpsL-erm*	CSS-0476	26695 Str^{R}	Str^{R} Erm^{R}
[a]***tlpB*::3xFLAG*/ *tlpB* 11G**	*rpsL-str^{R}*, *tlpB*::*tlpB*-3xFLAG*, *tlpB* 11G::*tlpB*:*rpsL-erm*	CSS-0477	26695 Str^{R}	Str^{R} Erm^{R}
[a]***tlpB*::3xFLAG*/ *tlpB* WT (12G)**	*rpsL-str^{R}*, *tlpB*::*tlpB*-3xFLAG*, *tlpB* 12G::*tlpB*:*rpsL-erm*	CSS-0470	26695 Str^{R}	Str^{R} Erm^{R}
[a]***tlpB*::3xFLAG*/ *tlpB* 13G**	*rpsL-str^{R}*, *tlpB*::*tlpB*-3xFLAG*, *tlpB* 13G::*tlpB*:*rpsL-erm*	CSS-0478	26695 Str^{R}	Str^{R} Erm^{R}
[a]***tlpB*::3xFLAG*/ *tlpB* 14G**	*rpsL-str^{R}*, *tlpB*::*tlpB*-3xFLAG*, *tlpB* 14G::*tlpB*:*rpsL-erm*	CSS-0479	26695 Str^{R}	Str^{R} Erm^{R}
[a]***tlpB*::3xFLAG*/ *tlpB* 15G**	*rpsL-str^{R}*, *tlpB*::*tlpB*-3xFLAG*, *tlpB* 15G::*tlpB*:*rpsL-erm*	CSS-0480	26695 Str^{R}	Str^{R} Erm^{R}
[a]***tlpB*::3xFLAG*/ *tlpB* 16G**	*rpsL-str^{R}*, *tlpB*::*tlpB*-3xFLAG*, *tlpB* 16G::*tlpB*:*rpsL-erm*	CSS-0481	26695 Str^{R}	Str^{R} Erm^{R}
[a]***tlpB*::3xFLAG*/ Δ*repG* / *tlpB* ΔG**	*rpsL-str^{R}*, *tlpB*::*tlpB*-3xFLAG*, *repG*::*aphA*-3, *tlpB* ΔG::*tlpB*:*rpsL-erm*	CSS-0483	26695 Str^{R}	Str^{R} Erm^{R} Kan^{R}

[a]tlpB::3xFLAG*/ ΔrepG / tlpB 6G	rpsL-str^R, tlpB::tlpB-3xFLAG*, repG::aphA-3, tlpB 6G::tlpB:rpsL-erm	CSS-0484	26695 Str^R	Str^R Erm^R Kan^R
[a]tlpB::3xFLAG*/ ΔrepG / tlpB 7G	rpsL-str^R, tlpB::tlpB-3xFLAG*, repG::aphA-3, tlpB 7G::tlpB:rpsL-erm	CSS-0485	26695 Str^R	Str^R Erm^R Kan^R
[a]tlpB::3xFLAG*/ ΔrepG / tlpB 8G	rpsL-str^R, tlpB::tlpB-3xFLAG*, repG::aphA-3, tlpB 8G::tlpB:rpsL-erm	CSS-0486	26695 Str^R	Str^R Erm^R Kan^R
[a]tlpB::3xFLAG*/ ΔrepG / tlpB 9G	rpsL-str^R, tlpB::tlpB-3xFLAG*, repG::aphA-3, tlpB 9G::tlpB:rpsL-erm	CSS-0487	26695 Str^R	Str^R Erm^R Kan^R
[a]tlpB::3xFLAG*/ ΔrepG / tlpB 10G	rpsL-str^R, tlpB::tlpB-3xFLAG*, repG::aphA-3, tlpB 10G::tlpB:rpsL-erm	CSS-0488	26695 Str^R	Str^R Erm^R Kan^R
[a]tlpB::3xFLAG*/ ΔrepG / tlpB 11G	rpsL-str^R, tlpB::tlpB-3xFLAG*, repG::aphA-3, tlpB 11G::tlpB:rpsL-erm	CSS-0489	26695 Str^R	Str^R Erm^R Kan^R
[a]tlpB::3xFLAG*/ ΔrepG / tlpB WT (12G)	rpsL-str^R, tlpB::tlpB-3xFLAG*, repG::aphA-3, tlpB 12G::tlpB:rpsL-erm	CSS-0482	26695 Str^R	Str^R Erm^R Kan^R
[a]tlpB::3xFLAG*/ ΔrepG / tlpB 13G	rpsL-str^R, tlpB::tlpB-3xFLAG*, repG::aphA-3, tlpB 13G::tlpB:rpsL-erm	CSS-0490	26695 Str^R	Str^R Erm^R Kan^R
[a]tlpB::3xFLAG*/ ΔrepG / tlpB 14G	rpsL-str^R, tlpB::tlpB-3xFLAG*, repG::aphA-3, tlpB 14G::tlpB:rpsL-erm	CSS-0491	26695 Str^R	Str^R Erm^R Kan^R
[a]tlpB::3xFLAG*/ ΔrepG / tlpB 15G	rpsL-str^R, tlpB::tlpB-3xFLAG*, repG::aphA-3, tlpB 15G::tlpB:rpsL-erm	CSS-0492	26695 Str^R	Str^R Erm^R Kan^R
[a]tlpB::3xFLAG*/ ΔrepG / tlpB 16G	rpsL-str^R, tlpB::tlpB-3xFLAG*, repG::aphA-3, tlpB 16G::tlpB:rpsL-erm	CSS-0493	26695 Str^R	Str^R Erm^R Kan^R
tlpB-hp0102 10th::gfpmut3	rdxA::tlpB-hp0102 10th-gfpmut3:catGC	CSS-2104	G27	Cm^R
tlpB-hp0102 10th::gfpmut3 / ΔrepG	repG::aphA-3, rdxA::tlpB-hp0102 10th-gfpmut3:catGC	CSS-2107	G27	Cm^R Kan^R
tlpB$_{mini}$-hp0102 10th::gfpmut3	rdxA::tlpB$_{mini}$-hp0102 10th-gfpmut3:catGC	CSS-2116	G27	Cm^R
tlpB$_{mini}$-hp0102 10th::gfpmut3 / ΔrepG	repG::aphA-3, rdxA::tlpB$_{mini}$-hp0102 10th-gfpmut3:catGC	CSS-2119	G27	Cm^R Kan^R
P$_{tlpB}$hp0102 10th::gfpmut3	rdxA::P$_{tlpB}$hp0102 10th-gfpmut3:catGC	CSS-2138	G27	Cm^R
P$_{tlpB}$hp0102 10th::gfpmut3 / ΔrepG	repG::aphA-3, rdxA::P$_{tlpB}$hp0102 10th-gfpmut3:catGC	CSS-2141	G27	Cm^R Kan^R
tlpB-hp0102 10th::gfpmut3 ΔG^{hp0102}	rdxA::tlpB-hp0102 10th-gfpmut3 ΔG^{hp0102}:catGC	CSS-2150	G27	Cm^R
tlpB-hp0102 10th::gfpmut3 ΔG^{hp0102} / ΔrepG	repG::aphA-3, rdxA::tlpB-hp0102 10th-gfpmut3 ΔG^{hp0102}:catGC	CSS-2153	G27	Cm^R Kan^R
tlpB-hp0102 10th::gfpmut3 ATTTA^{hp0102}	rdxA::tlpB-hp0102 10th-gfpmut3 ATTTA^{hp0102}:catGC	CSS-2156	G27	Cm^R
tlpB-hp0102 10th::gfpmut3 ATTTA^{hp0102} / ΔrepG	repG::aphA-3, rdxA::tlpB-hp0102 10th-gfpmut3 ATTTA^{hp0102}:catGC	CSS-2159	G27	Cm^R Kan^R
tlpB$_{stop}$-hp0102 10th::gfpmut3	rdxA::tlpB$_{stop}$-hp0102 10th-gfpmut3:catGC	CSS-3241	G27	Cm^R
tlpB$_{stop}$-hp0102 10th::gfpmut3/ ΔrepG	repG::aphA-3, rdxA::tlpB$_{stop}$-hp0102 10th-gfpmut3:catGC	CSS-3244	G27	Cm^R Kan^R
X47-2AL	Wildtype (DDBJ/EMBL/GenBank Acc-no. AWNG00000000, AWNG01000000); Ref[13]	CSS-0996	X47-2AL	
X47-2AL ΔrepG	repG::aphA-3	CSS-0997	X47-2AL	Kan^R
X47-2AL ΔtlpB	tlpB::aac(3)-IV	CSS-1123	X47-2AL	Gen^R
X47-2AL ΔtlpB / ΔrepG	tlpB::aac(3)-IV, repG::aphA-3	CSS-1769	X47-2AL	Gen^R Kan^R
X47-2AL ΔtlpB-hp0102	tlpB-hp0102::aac(3)-IV	CSS-1743	X47-2AL	Gen^R
X47-2AL ΔtlpB-hp0102 / ΔrepG	tlpB-hp0102::aac(3)-IV, repG::aphA-3	CSS-1773	X47-2AL	Gen^R Kan^R
X47-2AL ΔtlpB-hp0102 + tlpB-hp0102	tlpB-hp0102::aac(3)-IV, rdxA::tlpB-hp0102:aphA-3	CSS-2046	X47-2AL	Gen^R Kan^R
X47-2AL ΔtlpB-hp0102 + hp0102	tlpB-hp0102::aac(3)-IV, rdxA::hp0102:aphA-3	CSS-2080	X47-2AL	Gen^R Kan^R
X47-2AL Δhp0102	hp0102::aac(3)-IV	CSS-2019	X47-2AL	Gen^R
X47-2AL Δhp0102 / ΔrepG	hp0102::aac(3)-IV, repG::aphA-3	CSS-2022	X47-2AL	Gen^R Kan^R
X47-2AL Δhp0102 + hp0102	hp0102::aac(3)-IV, rdxA:: hp0102:aphA-3	CSS-2087	X47-2AL	Gen^R Kan^R

**X47-2AL Δ*hp1284***	*hp1284*::*aac*(3)-IV	CSS-5910	X47-2AL	Gen^R
**X47-2AL Δ*hp1039***	*hp1039*::*aac*(3)-IV	CSS-5912	X47-2AL	Gen^R
**X47-2AL Δ*hp1581***	*hp1581*::*aac*(3)-IV	CSS-5914	X47-2AL	Gen^R
**X47-2AL Δ*hp0159***	*hp0159*::*aac*(3)-IV	CSS-5916	X47-2AL	Gen^R
**X47-2AL Δ*hp0826***	*hp0826*::*aac*(3)-IV	CSS-5918	X47-2AL	Gen^R
**X47-2AL Δ*hp0579-580***	*hp0579-580*::*aac*(3)-IV	CSS-5920	X47-2AL	Gen^R
**X47-2AL Δ*hp1206***	*hp1206*::*aac*(3)-IV	CSS-5922	X47-2AL	Gen^R
**G27**	Wildtype (NCBI Acc-no. NC_011333), kindly provided by T. F. Meyer; Ref[14]	CSS-0010	G27	
[a]**G27 Δ*repG***	*repG*::*aphA*-3	CSS-0169	G27	Kan^R
**G27 Δ*hp0102***	*hp0102*::*rpsL-erm*	CSS-1007	G27	Erm^R
**J99**	Wildtype (NCBI Acc-no. NC_000921), kindly provided by T. F. Meyer, MPI-IB, Berlin, Germany; Ref[15]	CSS-0001	J99	
[a]**J99 Δ*repG***	*repG*::*aphA*-3	CSS-0732	J99	Kan^R
**J99 Δ*hp0102***	*hp0102*::*rpsL-erm*	CSS-1019	J99	Erm^R
**TOP 10**	mcrA Δ(mrr-hsdRMS-mcrBC) Φ80lacZΔM15 ΔlacX74recA1araD139 Δ(ara-leu)7697galUgalK λ⁻rpsLendA1nupG from Invitrogen	CSO-0296	*E. coli*	

Str^R: streptomycin resistant; Kan^R: kanamycin resistant; Cm^R: chloramphenicol resistant; Erm^R: erythromycin resistant; Gen^R: gentamicin resistant

[a] Mutant strains were constructed as described in Ref[2]

Supplementary Table 5: DNA oligonucleotides. Sequences are given in 5' –> 3' direction; P~ denotes a 5' monophosphate.

**Name**	**Sequence 5' -> 3'**	**Description**
**CSO-0003**	GAAAGGAGGGGGAGGT	Northern blot probe for RepG
**CSO-0017**	gttttTCTAGAGATCAGCCTGCCTTTAGG	RepG cloning
**CSO-0018**	gttttCTCGAGCTTAGCGCTTAATGAAACGC	RepG cloning
**CSO-0039**	gttttCTCGAGTCTCAAAATCCGCTGAAATCT	Cloning of *tlpB*
**CSO-0040**	gttttTCTAGATCAGTTGCAACCAGGAGATT	Cloning of *tlpB*
**CSO-0051**	TGTCACTTATATTTACAAGTTCGCT	Verification of *tlpB* deletion
**CSO-0086**	gttttATGCATAGGACTTTGAATGTCAAATCCATT	Verification/Sequencing of *tlpB-hp0102*, *hp0102*
**CSO-0146**	gttttATCGATGTATGCTCTTTAAGACCCAGC	Cloning GFP fusion, *tlpB-hp0102*
**CSO-0147**	gttttCATATGCTCGAATTCAGATCCACGTT	Cloning GFP fusion, *tlpB-hp0102*
**CSO-0205**	AATTACAACAGTACTGCGATGAGT	Verification/Sequencing of GFP fusions
**CSO-0206**	AATCTCACGCCAAGCATTT	Verification/Sequencing of *tlpB-hp0102*, *hp0102*
**CSO-0207**	AGTTCTGATTTCATGCCCTT	Verification/Sequencing of *tlpB-hp0102*, *hp0102*
**CSO-0263**	GACTGATGTCATCAGCGGT	Cloning of *tlpB*
**CSO-0293**	gttttGAATTcTCCAACGTCATCTCGTTCT	Cloning of *tlpB*, *tlpB-hp0102*
**CSO-0309**	gttttGAATTCTTACTTATTAAATAATTTATAGCTATTGAAAAGA	Cloning of *tlpB, hp0102*
**CSO-0581**	gttttATCGATTTATTATTTTATCTTTAAGCCTAACTTAA	Cloning of GFP fusion
**CSO-0683**	gttttTGCTAGCAGTAAAGGAGAAGAACTTTTCACTGGA	Cloning of GFP fusion
**CSO-0867**	AGCCATTTATGAACGCTACGGC	Quantitative RT-PCR (*hp0102*)
**CSO-0868**	CACCACAAACAAGCGGATGAT	Quantitative RT-PCR (*hp0102*)
**CSO-0869**	gttttGGATCCAACCCTTTTTTAATAAACTCC	Cloning *hp0102*
**CSO-0870**	gttttCTCGAGTTATCAATGATATTGCCGATCAAAC	Cloning *hp0102*
**CSO-0871**	gttttGAATTCCATGCTTTTAAAATACCCTAG	Cloning *hp0102*, *tlpB-hp0102*
**CSO-0872**	gttttTCTAGATTTGGATACAACTATTCGCAAGC	Cloning *hp0102*
**CSO-0873**	gttttTCTAGAGGCATCAAATAAAACGAAA	Cloning *hp0102*
**CSO-0874**	gttttCTCGAGGTGAAGACGAAAGG	Cloning *hp0102*, *tlpB-hp0102*
**CSO-0940**	gttttGGATCCCAGCGATTTCTTCTAATTCTGT	Cloning *tlpB-hp0102*
**CSO-0941**	gttttGGATCCACAAGCTGAAATTATAGAACACC	Cloning *tlpB-hp0102*
**CSO-0942**	gttttGAATTCTCAGGGGGAGTTTATTAAAAAAG	Cloning of *tlpB*
**CSO-1173**	GGTAGTGGTTTTTGTGTGATGG	Quantitative RT-PCR (6S RNA)
**CSO-1174**	CCAGATGACCGCTACTTTTACA	Quantitative RT-PCR (6S RNA)
**CSO-1359**	gttttCTCGAGTTTGGATACAACTATTCGCAAGC	Cloning *hp0102*, *tlpB-hp0102*

**CSO-1737**	gttttTCTAGATTATCAATGATATTGCCGATCAAAC	Cloning *hp0102*
**CSO-1738**	TGTCAAAGAGAGCAAGGATGC	Verification of *hp0102* deletion
**CSO-1739**	gttttGGATCCAACACGCCGTGATCACAGAA	Cloning *hp0102*
**CSO-1740**	gttttCATATGTAAGATTTTTTATTTTATTTTTAAGCCTAAC	Cloning *tlpB-hp0102*
**CSO-1741**	gttttATCGATAGTTAGGGATTGAAATACCCTTA	Cloning *tlpB-hp0102*
**CSO-1742**	AGCTAAAATTATAGAACACCCTTTT	Cloning *hp0102*
**CSO-1743**	P~TCAGGGGGAGTTTATCAAAAAAG	Cloning *hp0102*
**CSO-1745**	CATTTATCTCCTATAAATCATTTTTAAGT	Cloning of *tlpB*
**CSO-1803**	gttttGCTAGCACACGCCGTGATCACAGAAA	Cloning of GFP fusion
**CSO-1813**	gttttGGATCCTTTTATGGATAATTTTTAAAATCATTTG	Cloning *tlpB-hp0102*
**CSO-1814**	gttttGCTAGCTATTCCCTCCAGGTACTAAAACA	Cloning *tlpB-hp0102*
**CSO-1984**	GGCTAACACGACCAGCATGA	Cloning of GFP fusion
**CSO-1985**	P~TAATCAGGGGGAGTTTATTAAAAAAG	Cloning of GFP fusion
**CSO-2052**	P~AGTTTATTAAAAAAGGGTTGGATTG	Cloning of GFP fusion
**CSO-2053**	TGATTAAGTTTTAAACAAATTCACTTGT	Cloning of GFP fusion
**CSO-2055**	P~TCAGGGGGAGTTTATTAAAAAAGG	Cloning of GFP fusion
**CSO-2056**	AGCTGAAATTATAGAACACCCTTTTT	Cloning of GFP fusion
**CSO-2061**	P~ATTTAAGTTTATTAAAAAAGGGTTGGATTG	Cloning of GFP fusion
**CSO-2274**	TTGAAGAAAATTACTATTATCTCCTATAAATCATTTTTAAGTCAAA	Cloning of GFP fusion
**CSO-2275**	TAGGAGATAATAGTAATTTTCTTCAATGTTTGCTTCGTTG	Cloning of GFP fusion
**CSO-3195**	gttttGGATCCGACTGATGTCATCAGCGGT	Cloning *hp0102*
**CSO-SP001**	TCGCGGCTATAACTTTGATATG	Cloning of *tlpB* (Kan) in 26695
**CSO-SP004**	CCATAAAGCAAGGTTTTAGGGT	Cloning of *tlpB* (Kan) in 26695
**CSO-SP008**	TAGAGATCCGCCATATTGTGTT	Amplification of *aac*(3)-IV cassette
**CSO-SP009**	TCCAACGTCATCTCGTTCTC	Amplification of *aac*(3)-IV cassette
**CSO-SP010**	ATGGATTTTATAGGGTTTGAAGATTT	Cloning of *hp1284* in 26695 and X47
**CSO-SP013**	AATCAAACCCATATCGTTTGAGC	Cloning of *hp1284* in 26695 and X47
**CSO-SP014**	GCCCATAAAAGCATGGCAGA	Verification of *hp1284* deletion in 26695 and X47
**CSO-SP015**	ATGGCTCACTCTGCTTTTTTGG	Cloning of *hp1039* in 26695 and X47
**CSO-SP018**	ATCATTCCTACAAAAAAACTTCCC	Cloning of *hp1039* in 26695 and X47
**CSO-SP019**	CTTTAACTCAAGTCTTGTTGATTG	Verification of *hp1039* deletion in 26695 and X47
**CSO-SP020**	ATGCTTTTTGTGGATAACGCTAAT	Cloning of *hp1581* in 26695 and X47
**CSO-SP023**	CATCGCCAATCTCTTCTAAAATG	Cloning of *hp1581* in 26695 and X47
**CSO-SP024**	GCTATAATAGTGAAAATACTAAATCCA	Verification of *hp1581* deletion in 26695 and X47
**CSO-SP025**	TTGCGTGTTTTTGCCATTTCTTTAA	Cloning of *hp0826* in 26695 and X47

**CSO-SP028**	GAAGCTAATCCTAAAAAGTCTTAGT	Cloning of *hp0826* in 26695 and X47
**CSO-SP029**	GTTTTTAGTTGGATATTTAATCATCAAATT	Verification of *hp0826* deletion in 26695 and X47
**CSO-SP030**	ATGAGTATTATTATTCCTATTGTCATC	Cloning of *hp0159* in 26695 and X47
**CSO-SP033**	CGTTTGAAAATCCTATTCAATCTAAA	Cloning of *hp0159* in 26695 and X47
**CSO-SP034**	ACCCCAATCTAATTGGTTATTATAG	Verification of *hp0159* deletion in 26695 and X47
**CSO-SP035**	ATCTTGGAACCTTCAAGAAATCG	Cloning of *hp0579-580* in 26695 and X47
**CSO-SP038**	ACAACCTTTTTTTAACAAGAGATAAAG	Cloning of *hp0579-580* in 26695 and X47
**CSO-SP039**	TGCCCAGTAAAAAAGCGCGTT	Verification of *hp0579-580* deletion in 26695 and X47
**CSO-SP040**	CTACTTTAAAATACTTTTTGCGCTC	Cloning of *hp1206* in 26695 and X47
**CSO-SP043**	TTAGCCGAGATTGTCTTTGTGTT	Cloning of *hp1206* in 26695 and X47
**CSO-SP044**	TAGCTATAATCTAGCTCAATTTGG	Verification of *hp1206* deletion in 26695 and X47
**CSO-SP047**	AACACAATATGGCGGATCTCTAGTTAGGGAGGTTGTGATCTTTA	Cloning of *hp1284* in X47
**CSO-SP048**	GAGAACGAGATGACGTTGGAAATCGCATGGAAACTTAAAGACAA	Cloning of *hp1284* in X47
**CSO-SP049**	AACACAATATGGCGGATCTCTACCAATGGTTTGAGCGATTTTTTG	Cloning of *hp1039* in X47
**CSO-SP050**	GAGAACGAGATGACGTTGGAGTTATATGGTGGTCGTGAGTC	Cloning of *hp1039* in X47
**CSO-SP051**	AACACAATATGGCGGATCTCTACCTAAAAAAATCTTTCCTAAAGGGA	Cloning of *hp1581* in X47
**CSO-SP052**	GAGAACGAGATGACGTTGGATATTTTTTGGGTTTGGTGTGCG	Cloning of *hp1581* in X47
**CSO-SP053**	AACACAATATGGCGGATCTCTATATCTCGTGGTTTTTATGGCTC	Cloning of *hp0826* in X47
**CSO-SP054**	GAGAACGAGATGACGTTGGACAAGAGCGTGTGGGGATCA	Cloning of *hp0826* in X47
**CSO-SP055**	AACACAATATGGCGGATCTCTAGCCGCTCCAAAATAATAGCC	Cloning of *hp0159* in X47
**CSO-SP056**	GAGAACGAGATGACGTTGGATCCCTTTATGAGCATTACCTTAAT	Cloning of *hp0159* in X47
**CSO-SP057**	AACACAATATGGCGGATCTCTACAAACTTAAAACGAATCGGCTCT	Cloning of *hp0579-580* in X47
**CSO-SP058**	GAGAACGAGATGACGTTGGATCAAAAATAAGCCATTGGGTTTGA	Cloning of *hp0579-580* in X47
**CSO-SP059**	AACACAATATGGCGGATCTCTAGGAGTAGAAAAAAACGATCACAG	Cloning of *hp1206* in X47
**CSO-SP060**	GAGAACGAGATGACGTTGGAAGCGGATATTATTATGGGGCTTA	Cloning of *hp1206* in X47
**CSO-SP061**	ATTCTTCAGATATTTTAAACATCGCTAC	RT-PCR (*tlpB-hp0102*)
**CSO-SP062**	TATAAGTTTGATGAAGCACGGAAAGA	RT-PCR (*tlpB-hp0102*)
**CSO-SP063**	TCCTAGTTAGTCACCCGGGTAAAACAAGCTGAAATTATAGAACACC	Cloning of *tlpB-hp0102* in 26695
**CSO-SP064**	ATTGTTTTAGTACCTGGAGGGAATATGGAACGACCATGAATTTAGAC	Cloning of *tlpB-hp0102* in 26695
**CSO-SP065**	ATTGTTTTAGTACCTGGAGGGAATAATGAGTGAAAAAGATGAGGGCAT	Cloning of *hp0102* in 26695
**CSO-SP066**	TCCTAGTTAGTCACCCGGGTAGTTAGGGAGGTTGTGATCTTTA	Cloning of *hp1284* in 26695
**CSO-SP067**	ATTGTTTTAGTACCTGGAGGGAATAAATCGCATGGAAACTTAAAGACAA	Cloning of *hp1284* in 26695
**CSO-SP068**	TCCTAGTTAGTCACCCGGGTACCAATGGTTTGAGCGATTTTTTG	Cloning of *hp1039* in 26695
**CSO-SP069**	ATTGTTTTAGTACCTGGAGGGAATAGTTATATGGTGGTCGTGAGTC	Cloning of *hp1039* in 26695
**CSO-SP070**	TCCTAGTTAGTCACCCGGGTACCTAAAAAAATCTTTCCTAAAGGGA	Cloning of *hp1581* in 26695

**CSO-SP071**	ATTGTTTTAGTACCTGGAGGGAATATATTTTTTGGGTTTGGTGTGCG	Cloning of *hp1581* in 26695
**CSO-SP072**	TCCTAGTTAGTCACCCGGGTATATCTCGTGGTTTTTATGGCTC	Cloning of *hp0826* in 26695
**CSO-SP073**	ATTGTTTTAGTACCTGGAGGGAATACAAGAGCGTGTGGGGATCA	Cloning of *hp0826* in 26695
**CSO-SP074**	TCCTAGTTAGTCACCCGGGTAGCCGCTCCAAAATAATAGCC	Cloning of *hp0159* in 26695
**CSO-SP075**	ATTGTTTTAGTACCTGGAGGGAATATCCCTTTATGAGCATTACCTTAAT	Cloning of *hp0159* in 26695
**CSO-SP076**	TCCTAGTTAGTCACCCGGGTACAAACTTAAAACGAATCGGCTCT	Cloning of *hp0579-580* in 26695
**CSO-SP077**	ATTGTTTTAGTACCTGGAGGGAATATCAAAAATAAGCCATTGGGTTTGA	Cloning of *hp0579-580* in 26695
**CSO-SP078**	TCCTAGTTAGTCACCCGGGTAGGAGTAGAAAAAAACGATCACAG	Cloning of *hp1206* in 26695
**CSO-SP079**	ATTGTTTTAGTACCTGGAGGGAATAAGCGGATATTATTATGGGGCTTA	Cloning of *hp1206* in 26695
**CSONIH-0033**	TCAAAGCCACTAGTAAGTCTTACTT	Verification oligo for insertion of *rpsL-erm* cassette
**JVO-0155**	CCGTATGTAGCATCACCTTC	Cloning of GFP fusion [1616]
**JVO-0485**	TCGGAATGGTTAACTGGGTAGTTCCT	Northern blot probe for *Helicobacter pylori* 5S rRNA
**JVO-2134**	AAACCATAAGGAATGGTTGGAT	Northern blot probe for RepG
**JVO-5069**	CTTCACGCCCCTTGTAAATA	Verification of *repG* deletion mutant
**JVO-5070**	GATAAGGTTTAGCGATGTAATCGT	RepG cloning
**JVO-5072**	CGTTTCTTGACACGCTTAATT	RepG cloning
**JVO-5267**	ACGGGGTGGTATTGTTTGAT	Quantitative RT-PCR (*tlpB*)
**JVO-5268**	AAGTGTAGCCTCCCCCTTTT	Quantitative RT-PCR (*tlpB*)
**JVO-5257**	TATAGGTTTTCATTTTCTCCCAC	Verification of *repG* deletion mutant
**pZE-A**	GTGCCACCTGACGTCTAAGA	Colony PCR and sequencing of pZE12-*luc* derived plasmids
**pZE-*XbaI***	TCGTTTTATTTGATGCCTCTAGA	Colony PCR and sequencing of pZE derived plasmids
**HPK-1**	GTACCCGGGTGACTAACTAGG	Amplification of *aphA-3* cassette
**HPK-2**	TATTCCCTCCAGGTACTAAAACA	Amplification of *aphA-3* cassette

Supplementary Table 6: Plasmids

Name	Description/Generation	Origin / marker	Reference
**pBA1-1**	Intermediary plasmid for construction of pBA7-4	p15A/ Amp^R Erm^R	This study
**pBA4-2**	Intermediary plasmid for construction of pSP189-4 (pSP190-1)	p15A/ Amp^R Gen^R	This study
**pBA5-4**	Intermediary plasmid for construction of pBA13-5	p15A/ Amp^R	This study
**pBA7-4**	Plasmid for deletion of *hp0102* in diverse *H. pylori* strains	p15A/ Amp^R Erm^R	This study
**pBA13-5**	Plasmid for deletion of *tlpB* in *H. pylori* strain X47-2AL	p15A/ Amp^R Gen^R	This study
**pJV752-1**	Cloning vector, pZE12-*luc* with modified p15A origin	p15A/ Amp^R	Ref[17]
**pMA5-2**	Plasmid for translational fusion of the *cagA*-5' UTR including the 28th amino acid to *gfpmut3*	p15A/ Amp^R Cm^R	Ref[2]
**pSP39-3**	Plasmid for complementation of *repG* deletion with RepG in *H. pylori* strain 26695	p15A/ Amp^R Cm^R	Ref[2]
**pSP60-2**	Backbone plasmid for deletion or nucleotide exchange in G-stretch in 5' UTR of *tlpB*	p15A/ Amp^R Erm^R	Ref[2]
**pSP127-3**	Plasmid for deletion of the *tlpB-hp0102* operon in *H. pylori* strain X47-2AL	p15A/ Amp^R Gen^R	This study
**pSP186-2**	Plasmid for deletion of *hp0102* in *H. pylori* strain X47-2AL	p15A/ Amp^R Gen^R	This study
**pSP189-4**	Intermediary plasmid for construction of pSP190-1	p15A/ Amp^R Gen^R	This study
**pSP190-1**	Plasmid for complementation of the *H. pylori* X47-2AL Δ*tlpB-hp0102* mutant with the *tlpB-hp0102* operon	p15A/ Amp^R Kan^R	This study
**pSP192-1**	Plasmid for complementation of the *H. pylori* X47-2AL Δ*tlpB-hp0102* and Δ*hp0102* mutants with *hp0102* alone	p15A/ Amp^R Kan^R	This study
**pSP195-6**	Plasmid for construction of *tlpB-hp0102* 10th::*gfpmut3* reporter fusion	p15A/ Amp^R Cm^R	This study
**pSP197-3**	Plasmid for construction of $tlpB_{mini}$-*hp0102* 10th::*gfpmut3* reporter fusion	p15A/ Amp^R Cm^R	This study
**pSP198-4**	Plasmid for construction of P_{tlpB}*hp0102* 10th::*gfpmut3* reporter fusion	p15A/ Amp^R Cm^R	This study
**pSP200-2**	Plasmid for construction of *tlpB-hp0102* 10th::*gfpmut3* ΔG^{hp0102} reporter fusion	p15A/ Amp^R Cm^R	This study
**pSP201-1**	Plasmid for construction of *tlpB-hp0102* 10th::*gfpmut3* $ATTTA^{hp0102}$ reporter fusion	p15A/ Amp^R Cm^R	This study
**pSP205-17**	Plasmid for construction of $tlpB_{stop}$-*hp0102* 10th::*gfpmut3* reporter fusion	p15A/ Amp^R Cm^R	This study
**pUC1813apra**	Plasmid carrying apramycin/gentamicin resistance cassette *aac*(3)-IV	pMB1/ Amp^R Gen^R	Ref[18]

Kan^R: kanamycin resistant; Cm^R: chloramphenicol resistant; Erm^R: erythromycin resistant; Gen^R: gentamicin resistant

Supplementary Table 7: Sequences of *tlpB-hp0102*::*gfpmut3* reporter fusions used in this study. The homopolymeric G-repeat in the 5' UTR of *tlpB* mRNA and the G^{hp0102}-repeat in the *tlpB-hp0102* IGR are underlined. The RBSs as well as start codons and ORFs are shown in light green and gray letters, respectively. Mutations within the G^{hp0102}-repeat are shown in red. Alteration of the *tlpB* start codon is shown in blue (two stop codons). The *NheI* restriction site is marked in yellow, the *gfpmut3* coding sequence is highlighted in green.

Name	Sequence 5' → 3'
tlpB-hp0102* 10th*-gfpmut3	**T**GTTTGTTCTTTTGTTTCGTTTTCAAACAACCGGGTTTTAATTTTGTTTTGTGCCACTCATTTTTCGGGGGGGGGGGGTGCATTTAGAAGCTAAACTCTAAAATTAGGGTTTGA<br>CTTAAAAATGATTTATAGGAGATAAATGATGTTTTCTTCAATGTTTGCTTCGTTGGGGACTCGTATCATGCTGGTCGTGTTAGCCGCTCTTTTAGGTTTAGGGGGCTTTTTAT<br>TGGTTTTGTAAAGGTTATGCAAAAAGATGTGTTAGCGCAACTCATGGAGCATTTAGAAACCGGGCAATACAAAAAGCGTGAAAAAACGCTCGCTTACATGACAAAAATTATTGA<br>ACAGGGCATTCATGAGTATTACAAAAATTTTGACAATGCTACTGCAAGAAAAATGGCGTTAGATTATTTCAAACGCATCAACGACGATAAGGGCATGATTTATATGGTGGTGGT<br>GGATAAAAACGGGGTGGTATTGTTTGATCCGGTCAATCCTAAAACCGTAGGCCAATCAGGGCTTGACGCTCAGAGCGTTGATGGGGTGTATTATGTTAGGGGGTATTTGGAGGC<br>GGCCAAAAAAGGGGGAGGCTACACTTATTATAAAATGCCTAAATACGATGGAGGCGTACCGGAGAAAAAATTCGCCTACTCGCATTATGATGAAGTTTCTCAAATGGTGATCGC<br>AACGACTTCCTATTACACTGACATTAACACAGAAAATAAAGCGATCAAAGAAGGCGTGAATAAGGTTTTTGATGAAAACACCACGAAATTATTCCTTTGGATACTGACAGCGAC<br>GATAGCGCTAGTGGTTTTGACGCTCATATACGCTAAATTAAGGATCGTGAAACGCATTGATGAACTGGTCCTTAAAATCAACGCTTTTAGCCGTGGGGATAAGGATTTGAGAGC<br>CAAAATTGATGTGGGTGATCGCAACGATGAAATCTCGCAAGTGGGCCGTGGGATCAATTTGTTTGTGGAAAACGCCCGCTTGATTATGGAAGAGATTAAAGGGATTTCCACCCT<br>CAATAAAACTTCAATGGATAAATTAGTCCAAATCACGCAAGAAACCCAAAAGAGCATGAAAGATTCCTCAACCACCCTAAATTCCGTGAAAAATAAAGCCACTGATATAGCGAG<br>CATGATGAATGCTTCCATAGAGCAATCTCAAGGGTTAAGGAAGCGTTTGATTGAAACGCAAGGGCTGGTCAAAGAGAGCAAGGATGCGATCGGGGATTTATTTTCTCAAATCAC<br>AGAGAGCGCGCACACTGAAGAGGAACTCTCTAGCAAAGTGGAGCAGCTAAGCCGTAACGCTGATGATGTCAAATCCATTCTGGATATTATCAATGATATTGCCGATCAAACGAA<br>TTTATTAGCCCTAAACGCTGCTATTGAAGCCGCAAGGGCTGGCGAGCATGGCAGAGGCTTTGCGGTGGTGGCTGATGAAGTTAGGAATTTAGCCGGGCGCACTCAAAAGTCTTT<br>AGCCGAAATCAATTCCACTATCATGGTGATTGTCCAAGAAATCAATGCCGTGAGTTCGCAAATGAATCTCAATTCGCAAAAAATGGAGCGTTTGAGCGATATGAGTAAAAGCGT<br>GCAAGAAACTTACGAAAAAATGAGTTCTAATTTAAGCTCAGTCGTGTCAGACAGCAATCAAAGCATGGACGATTACGCCAAATCCGGACACCAAATTGAAGTTATGGTAAGCGA<br>TTTTGCAGAGGTGGAAAAAGTGGCTTCTAAGACTTTAGCGGATTCTTCAGATATTTTAAACATCGCTACGCATGTGAGTGGAACGACCATGAATTTAGACAAACAAGTGAATTT<br>GTTTAAAACTTAATCAGGGGGAGTTTATTAAAAAAGGGTTGGATTGTTAAAAGTTTCTGTGATCACGGCGTGTGCTAGCAGTAAAGGAGAAGAACTTTTCACTGGAGTTGTCCC<br>AATTCTTGTTGAATTAGATGGTGATGTTAATGGGCACAAATTTTCTGTCAGTGGAGAGGGTGAAGGTGATGCAACATACGGAAAACTTACCCTTAAATTTATTTGCACTACTGG<br>AAAACTACCTGTTCCATGGCCAACACTTGTCACTACTTTCGGTTATGGTGTTCAATGCTTTGCGAGATACCCAGATCATATGAAACAGCATGACTTTTTCAAGAGTGCCATGCC<br>CGAAGGTTATGTACAGGAAAGAACTATATTTTTCAAAGATGACGGGAACTACAAGACACGTGCTGAAGTCAAGTTTGAAGGTGATACCCTTGTTAATAGAATCGAGTTAAAAGG<br>TATTGATTTTAAAGAAGATGGAAACATTCTTGGACACAAATTGGAATACAACTATAACTCACACAATGTATACATCATGGCAGACAAACAAAAGAATGGAATCAAAGTTAACTT<br>CAAAATTAGACACAACATTGAAGATGGAAGCGTTCAACTAGCAGACCATTATCAACAAAATACTCCAATTGGCGATGGCCCTGTCCTTTTACCAGACAACCATTACCTGTCCAC<br>ACAATCTGCCCTTTCGAAAGATCCCAACGAAAAGAGAGACCACATGGTCCTTCTTGAGTTTGTAACAGCTGCTGGGATTACACATGGCATGGATGAACTATACAAATAA
tlpB*$_{mini}$*-hp0102* 10th*-gfpmut3	**T**GTTTGTTCTTTTGTTTCGTTTTCAAACAACCGGGTTTTAATTTTGTTTTGTGCCACTCATTTTTCGGGGGGGGGGGGTGCATTTAGAAGCTAAACTCTAAAATTAGGGTTTGA<br>CTTAAAAATGATTTATAGGAGATAAATGATGTTTTCTTCAATGTTTGCTTCGTTGGGGACTCGTATCATGCTGGTCGTGTTAGCCTAATCAGGGGGAGTTTATTAAAAAAGGGT<br>TGGATTGTTAAAAGTTTCTGTGATCACGGCGTGTGCTAGCAGT-GFPMUT3-TAA
P*$_{tlpB}$*hp0102* 10th*-gfpmut3	**T**CAGGGGGAGTTTATTAAAAAAGGGTTGGATTGTTAAAAGTTTCTGTGATCACGGCGTGTGCTAGCAGT-GFPMUT3-TAA
***tlpB-hp0102* 10th*-gfpmut3* ΔG^{hp0102}**	**T**GTTTGTTCTTTTGTTTCGTTTTCAAACAACCGGGTTTTAATTTTGTTTTGTGCCACTCATTTTTCGGGGGGGGGGGGTGCATTTAGAAGCTAAACTCTAAAATTAGGGTTTGA<br>CTTAAAAATGATTTATAGGAGATAAATGATG-TLPB CODING REGION-TAATCAAGTTTATTAAAAAAGGGTTGGATTGTTAAA<br>AGTTTCTGTGATCACGGCGTGTGCTAGCAGT-GFPMUT3-TAA

***tlpB-hp0102* 10th-*gfpmut3* ATTTA^{hp0102}**	**T**GTTTGTTCTTTTGTTTCGTTTTCAAACAACCGGGTTTTAATTTTGTTTTGTGCCACTCATTTTTCGGGGGGGGGGGGTGCATTTAGAAGCTAAACTCTAAAATTAGGGTTTGACTTAAAAATGATTTATAGGAGATAAATGATG-TLPB CODING REGION-TAATCAATTTAAGTTTATTAAAAAAGGGTTGGATTGTTAAAAGTTTCTGTGATCACGGCGTGTGCTAGCAGT-GFPMUT3-TAA
tlpB*$_{stop}$-*hp0102* 10th-*gfpmut3	**T**GTTTGTTCTTTTGTTTCGTTTTCAAACAACCGGGTTTTAATTTTGTTTTGTGCCACTCATTTTTCGGGGGGGGGGGGTGCATTTAGAAGCTAAACTCTAAAATTAGGGTTTGACTTAAAAATGATTTATAGGAGATAATAGTAATTTTCTTCAATGTTTGCTTCGTTGGGGACTCGTATCATGCTGGTCGTGTTAGCCTAATCAGGGGGAGTTTATTAAAAAAGGGTTGGATTGTTAAAAGTTTCTGTGATCACGGCGTGTGCTAGCAGT-GFPMUT3-TAA

Supplementary Table 8: Sequences of *tlpB* leader mutants of *H. pylori* strain 26695. The length-variable homopolymeric G-repeat in the 5' UTR of the *tlpB* mRNA is shown in red, and the RBS as well as start codon are marked in light green.

Name	Sequence 5' → 3'
tlpB WT	**T**GTTTGTTCTTTTGTTTCGTTTTCAAACAACCGGGTTTTAATTTTGTTTTGTGCCACTCATTTTTCGGGGGGGGGGGGTGCATTTAGAAGCTAAACTCTAAAATTAGGGTTTGACTTAAAAATGATTTATAGGAGATAAATG
tlpB ΔG	**T**GTTTGTTCTTTTGTTTCGTTTTCAAACAACCGGGTTTTAATTTTGTTTTGTGCCACTCATTTTTCTGCATTTAGAAGCTAAACTCTAAAATTAGGGTTTGACTTAAAAATGATTTATAGGAGATAAATG
tlpB 6G	**T**GTTTGTTCTTTTGTTTCGTTTTCAAACAACCGGGTTTTAATTTTGTTTTGTGCCACTCATTTTTCGGGGGGTGCATTTAGAAGCTAAACTCTAAAATTAGGGTTTGACTTAAAAATGATTTATAGGAGATAAATG
tlpB 7G	**T**GTTTGTTCTTTTGTTTCGTTTTCAAACAACCGGGTTTTAATTTTGTTTTGTGCCACTCATTTTTCGGGGGGGTGCATTTAGAAGCTAAACTCTAAAATTAGGGTTTGACTTAAAAATGATTTATAGGAGATAAATG
tlpB 8G	**T**GTTTGTTCTTTTGTTTCGTTTTCAAACAACCGGGTTTTAATTTTGTTTTGTGCCACTCATTTTTCGGGGGGGGTGCATTTAGAAGCTAAACTCTAAAATTAGGGTTTGACTTAAAAATGATTTATAGGAGATAAATG
tlpB 9G	**T**GTTTGTTCTTTTGTTTCGTTTTCAAACAACCGGGTTTTAATTTTGTTTTGTGCCACTCATTTTTCGGGGGGGGGTGCATTTAGAAGCTAAACTCTAAAATTAGGGTTTGACTTAAAAATGATTTATAGGAGATAAATG
tlpB 10G	**T**GTTTGTTCTTTTGTTTCGTTTTCAAACAACCGGGTTTTAATTTTGTTTTGTGCCACTCATTTTTCGGGGGGGGGGTGCATTTAGAAGCTAAACTCTAAAATTAGGGTTTGACTTAAAAATGATTTATAGGAGATAAATG
tlpB 11G	**T**GTTTGTTCTTTTGTTTCGTTTTCAAACAACCGGGTTTTAATTTTGTTTTGTGCCACTCATTTTTCGGGGGGGGGGGTGCATTTAGAAGCTAAACTCTAAAATTAGGGTTTGACTTAAAAATGATTTATAGGAGATAAATG
tlpB 13G	**T**GTTTGTTCTTTTGTTTCGTTTTCAAACAACCGGGTTTTAATTTTGTTTTGTGCCACTCATTTTTCGGGGGGGGGGGGGTGCATTTAGAAGCTAAACTCTAAAATTAGGGTTTGACTTAAAAATGATTTATAGGAGATAAATG
tlpB 14G	**T**GTTTGTTCTTTTGTTTCGTTTTCAAACAACCGGGTTTTAATTTTGTTTTGTGCCACTCATTTTTCGGGGGGGGGGGGGGTGCATTTAGAAGCTAAACTCTAAAATTAGGGTTTGACTTAAAAATGATTTATAGGAGATAAATG
tlpB 15G	**T**GTTTGTTCTTTTGTTTCGTTTTCAAACAACCGGGTTTTAATTTTGTTTTGTGCCACTCATTTTTCGGGGGGGGGGGGGGGTGCATTTAGAAGCTAAACTCTAAAATTAGGGTTTGACTTAAAAATGATTTATAGGAGATAAATG
tlpB 16G	**T**GTTTGTTCTTTTGTTTCGTTTTCAAACAACCGGGTTTTAATTTTGTTTTGTGCCACTCATTTTTCGGGGGGGGGGGGGGGGTGCATTTAGAAGCTAAACTCTAAAATTAGGGTTTGACTTAAAAATGATTTATAGGAGATAAATG

Supplementary References

1. Sharma, C. M. *et al.* The primary transcriptome of the major human pathogen *Helicobacter pylori*. *Nature* **464,** 250–255 (2010).
2. Pernitzsch, S. R., Tirier, S. M., Beier, D. & Sharma, C. M. A variable homopolymeric G-repeat defines small RNA-mediated posttranscriptional regulation of a chemotaxis receptor in *Helicobacter pylori*. *Proc. Natl. Acad. Sci. USA* **111,** E501-10 (2014).
3. Li, H. *et al.* Lipopolysaccharide Structure and Biosynthesis in *Helicobacter pylori*. *Helicobacter* **21,** 445–461 (2016).
4. Li, H. *et al.* East-Asian *Helicobacter pylori* strains synthesize heptan-deficient lipopolysaccharide. *PLoS Genet.* **15,** e1008497 (2019).
5. Kennemann, L. *et al. Helicobacter pylori* genome evolution during human infection. *Proc. Natl. Acad. Sci. USA* **108,** 5033–5038 (2011).
6. Devi, S. H. *et al.* Genome of *Helicobacter pylori* strain 908. *J. Bacteriol.* **192,** 6488–6489 (2010).
7. Avasthi, T. S. *et al.* Genomes of two chronological isolates (*Helicobacter pylori* 2017 and 2018) of the West African *Helicobacter pylori* strain 908 obtained from a single patient. *J. Bacteriol.* **193,** 3385–3386 (2011).
8. Linz, B. *et al.* A mutation burst during the acute phase of *Helicobacter pylori* infection in humans and rhesus macaques. *Nat. Commun.* **5,** 4165 (2014).
9. Nell, S. *et al.* Genome and Methylome Variation in *Helicobacter pylori* With a *cag* Pathogenicity Island During Early Stages of Human Infection. *Gastroenterology* **154,** 612–623.e7 (2018).
10. Salaün, L., Ayraud, S. & Saunders, N. J. Phase variation mediated niche adaptation during prolonged experimental murine infection with *Helicobacter pylori*. *Microbiology (Reading, Engl.)* **151,** 917–923 (2005).
11. Behrens, W. *et al.* Role of energy sensor TlpD of *Helicobacter pylori* in gerbil colonization and genome analyses after adaptation in the gerbil. *Infect. Immun.* **81,** 3534–3551 (2013).
12. Tomb, J. F. *et al.* The complete genome sequence of the gastric pathogen *Helicobacter pylori*. *Nature* **388,** 539–547 (1997).
13. Veyrier, F. J., Ecobichon, C. & Boneca, I. G. Draft Genome Sequence of Strain X47-2AL, a Feline *Helicobacter pylori* Isolate. *Genome Announc.* **1,** (2013).
14. Baltrus, D. A. *et al.* The complete genome sequence of *Helicobacter pylori* strain G27. *J. Bacteriol.* **191,** 447–448 (2009).
15. Alm, R. A. & Trust, T. J. Analysis of the genetic diversity of *Helicobacter pylori*: the tale of two genomes. *J. Mol. Med.* **77,** 834–846 (1999).
16. Urban, J. H. & Vogel, J. Translational control and target recognition by *Escherichia coli* small RNAs *in vivo*. *Nucleic Acids Res.* **35,** 1018–1037 (2007).
17. Sharma, C. M., Darfeuille, F., Plantinga, T. H. & Vogel, J. A small RNA regulates multiple ABC transporter mRNAs by targeting C/A-rich elements inside and upstream of ribosome-binding sites. *Genes Dev.* **21,** 2804–2817 (2007).
18. Bury-Moné, S. *et al.* Presence of active aliphatic amidases in *Helicobacter* species able to colonize the stomach. *Infect. Immun.* **71,** 5613–5622 (2003).
