# Supplementary material for: Small RNA mediated gradual control of lipopolysaccharide biosynthesis affects antibiotic resistance in Helicobacter pylori
Source: Nat Commun. 2021 Jul 21;12:4433. doi: 10.1038/s41467-021-24689-2 (PMC8295292; doi:10.1038/s41467-021-24689-2)
Supplement: Supplementary file 2 — Reporting Summary [file 41467_2021_24689_MOESM2_ESM.pdf]

nature research

Corresponding author(s): Cynthia M. Sharma
Last updated by author(s): Jun 18, 2021

Reporting Summary

Nature Research wishes to improve the reproducibility of the work that we publish. This form provides structure for consistency and transparency in reporting. For further information on Nature Research policies, see our Editorial Policies and the Editorial Policy Checklist.

Statistics

For all statistical analyses, confirm that the following items are present in the figure legend, table legend, main text, or Methods section.

n/a	Confirmed	
☐	☒	The exact sample size (*n*) for each experimental group/condition, given as a discrete number and unit of measurement
☐	☒	A statement on whether measurements were taken from distinct samples or whether the same sample was measured repeatedly
☐	☒	The statistical test(s) used AND whether they are one- or two-sided<br>*Only common tests should be described solely by name; describe more complex techniques in the Methods section.*
☒	☐	A description of all covariates tested
☒	☐	A description of any assumptions or corrections, such as tests of normality and adjustment for multiple comparisons
☐	☒	A full description of the statistical parameters including central tendency (e.g. means) or other basic estimates (e.g. regression coefficient) AND variation (e.g. standard deviation) or associated estimates of uncertainty (e.g. confidence intervals)
☐	☒	For null hypothesis testing, the test statistic (e.g. *F*, *t*, *r*) with confidence intervals, effect sizes, degrees of freedom and *P* value noted<br>*Give P values as exact values whenever suitable.*
☒	☐	For Bayesian analysis, information on the choice of priors and Markov chain Monte Carlo settings
☒	☐	For hierarchical and complex designs, identification of the appropriate level for tests and full reporting of outcomes
☒	☐	Estimates of effect sizes (e.g. Cohen's *d*, Pearson's *r*), indicating how they were calculated

Our web collection on statistics for biologists contains articles on many of the points above.

Software and code

Policy information about availability of computer code

Data collection	BLAST (Basic Local Alignment Search Tool, NCBI, version BLAST+ 2.8.1) and SyntTax webserver (Oberto 2013) were used for biocomputational searches for repG, tlpB, and hp0102 homologs in diverse Helicobacter species (spp.) and other related Epsilonproteobacteria, including Campylobacter spp., Wolinella spp., Sulfuricurvum spp., Arcobacter spp., Sulfurospirillum spp., and Nautilia profundicola.
Data analysis	Statistical analyses were performed using GraphPad Prism 6.0. Western Blot densitometry analyses were conducted using AIDA Image Analysis Software (v5.0 SP1, build 1182, 2014; Raytest, Germany).

For manuscripts utilizing custom algorithms or software that are central to the research but not yet described in published literature, software must be made available to editors and reviewers. We strongly encourage code deposition in a community repository (e.g. GitHub). See the Nature Research guidelines for submitting code & software for further information.

Data

Policy information about availability of data

All manuscripts must include a data availability statement. This statement should provide the following information, where applicable:

- Accession codes, unique identifiers, or web links for publicly available datasets
- A list of figures that have associated raw data
- A description of any restrictions on data availability

All data supporting the findings of this study are available within the article and its supplementary information files. The source data underlying Figs. 1b, 2b, 3b, d, 4a, b, 5a, b, 6b, c, and Supplementary Figs. 1b, 2, 3, 5, 7b, c, 8, 9b, c, d are provided as a Source Data file. All data is available from the corresponding author upon reasonable request.

Field-specific reporting

Please select the one below that is the best fit for your research. If you are not sure, read the appropriate sections before making your selection.

- [x] Life sciences
- [ ] Behavioural & social sciences
- [ ] Ecological, evolutionary & environmental sciences

For a reference copy of the document with all sections, see nature.com/documents/nr-reporting-summary-flat.pdf

Life sciences study design

All studies must disclose on these points even when the disclosure is negative.

Sample size	In-vivo mouse studies:<br>For the mouse experiments, the sample size was the same as in our previous publications. Having seven mice per test condition allows the application of a statistical analysis using the Mann Whitney U test.<br>In-vitro analyses:<br>All western blots, northern blots, and LPS proteins gels (SDS-PAGE) are shown as representatives of at least three independent biological replicates. RT-qPCR experiments were performed in at least three biological replicates comprised of two technical replicates each. Salt stress experiments and quantifications of polymyxin B minimal inhibitory concentrations (MICs) are based on 2-3 independent biological replicates. All sample sizes were selected based on prior experience with similar experimental set ups to determine statistical significance as previously published for other studies from our lab.
Data exclusions	No data were excluded.
Replication	All in-vitro experiments were reprodcued at least 2-3 times. All replications were sucessful. All in-vivo experiments were performed with 7 mice infected with H. pylori strains and 4-5 mice for non-infected controls. The in vivo experiments were reproduced twice and all replications were successful.
Randomization	For in-vivo experiments: the mice used were all five weeks old NMRI-specific pathogen-free female mice and were randomly assigned to the different test groups.<br>For in-vitro experiments (e.g., northern and western blots), no randomization was required as corresponding samples were processed together.
Blinding	Sample blinding was not relevant as none of the readouts was subjective.

Reporting for specific materials, systems and methods

We require information from authors about some types of materials, experimental systems and methods used in many studies. Here, indicate whether each material, system or method listed is relevant to your study. If you are not sure if a list item applies to your research, read the appropriate section before selecting a response.

Materials & experimental systems

n/a	Involved in the study	
☐	☒	Antibodies
☒	☐	Eukaryotic cell lines
☒	☐	Palaeontology and archaeology
☐	☒	Animals and other organisms
☒	☐	Human research participants
☒	☐	Clinical data
☒	☐	Dual use research of concern

Methods

n/a	Involved in the study	
☒	☐	ChIP-seq
☒	☐	Flow cytometry
☒	☐	MRI-based neuroimaging

Antibodies

Antibodies used	The following primary antibodies were utilized in this study: GroEL (Sigma Aldrich, #G6532 RRID: AB_259939, Lot: 055M4779V); GFP (Roche, #11814460001, Lot: 11063100, clone: 7.1 and 13.1); FLAG (Sigma Aldrich, #F1804 RRID: AB_262044, Lot: 11891200, clone: M2); Lewis x (Calbiochem, #434631, Lot: D00136553); Lewis y (Merck, #434636, Lot: 3220381); TlpA22-antiserum (kindly provided by Karen Ottemann, University of California, Santa Cruz, CA).<br>The following secondary antibodies were utilized in this study: sheep@mouse (GE Healthcare, #RPN4201, Lot: 397127); goat@rabbit (GE Healthcare, #RPN4301 RRID: AB_2650489).

Validation	All primary antibodies have been validated by the manufacturers and previously used for the same applications: GroEL, GFP, and FLAG (Dugar et al., 2016; PMID: 27229370); Lewis x and y (Sakamoto et al., 1986; PMID: 3510728); TlpA22-antiserum (Pernitzsch et al., 2014; PMID: 24474799; Collins et al., 2016; PMID:27002127).

Animals and other organisms

Policy information about studies involving animals; ARRIVE guidelines recommended for reporting animal research

Laboratory animals	Five weeks old NMRI-specific pathogen-free female mice from Charles River Laboratories were used. The housing conditions of the mice were optimal to reduce their stress. This included half/half light/dark cycle, a constant temperature of 20-23°C, about 50% humidity, and ad libitum access to water and food. Before infection, the animals underwent an acclimation period of one week.
Wild animals	No wild animals were used.
Field-collected samples	None.
Ethics oversight	Experiments in mice were carried out in strict accordance with the recommendations in the Specific Guide for the Care and the Use of Laboratory Animals of the Institut Pasteur, according to the European Directive (2010/63/UE) and the corresponding French law on animal experimentation (Arrêtés de 1988). The protocol has been approved by the Committee of Central Animal Facility Board of the Institut Pasteur. To follow the new European directives, the project was approved by the CETEA, Comité d'éthique en Expérimentation Animale of the Institut Pasteur (#2013-0051) and by the Ministère de l'Enseignement Supérieur et de la recherche (#751501).

Note that full information on the approval of the study protocol must also be provided in the manuscript.
